# Cofeeding at rich clumped food patches in free-ranging dogs: social tolerance or scramble competition?

**DOI:** 10.1007/s00265-025-03590-8

**Published:** 2025-04-14

**Authors:** Andreas Berghänel, Martina Lazzaroni, Malgorzata Ferenc, Malgorzata Pilot, Ikhlass el Berbri, Sarah Marshall-Pescini, Friederike Range

**Affiliations:** 1 Domestication Lab, Konrad Lorenz Institute of Ethology, University of Veterinary Medicine Vienna, Vienna, Austria; 2 Department of Chemistry, Life Science and Environmental Sustainability, University of Parma, Parma, Italy; 3 Faculty of Biology, University of Gdańsk, Gdańsk, Poland; 4 Department of Pathology and Veterinary Public Health, Agronomic and Veterinary Institute Hassan II, Rabat, Morocco

**Keywords:** Socio-ecology, Priority of access, Feeding competition, Swamping

## Abstract

**Abstract:**

Animals are generally expected to monopolize food patches whenever possible. However, cofeeding within a defendable range occurs in many species, particularly at larger food patches, but the mechanism behind that remains underexplored. In theory, it could be due to multiple, mutually non-exclusive processes. First, larger food patches may saturate multiple top-ranking individuals, enabling cofeeding even under pure contest competition. Second, cofeeding may result from social tolerance where dominant individuals provide cofeeding concessions to certain subordinates. Third, cofeeding may result from prevailing scramble competition (i.e., indirect competition through patch exploitation) caused by large numbers of individuals that prevent monopolization ("swamping"). To investigate and differentiate between these mechanisms, we applied feeding tests to free-ranging dogs in Morocco. We provided them with a large food patch plus a varying number of small food patches. Although the small food patches were virtually always monopolized by single individuals, the dogs typically cofed in large and very dense feeding groups at the large food patches. Controlling for alternative explanations using multivariate statistics, we found that access to feeding groups was independently predicted by rank and social relationship strength, suggesting that contest competition and social tolerance play a role. However, aggression rates by top-rankers decreased with increasing feeding group size, suggesting decreasing monopolizability and increasing scramble competition. Our results underscore that social tolerance may not reduce competition but shifts it from contest to scramble competition. This can be due to active levelling, licensing more individuals access to the resource, but also to loss of control caused by swamping.

**Significance statement:**

Although animals are generally expected to fight for resources, they are sometimes observed to cofeed peacefully in large groups. Such peaceful cofeeding is typically ascribed to and taken as a measure of social tolerance, assuming that dominants overcome their impulse to monopolize and make concessions to lower-ranking group members. Alternatively, such large peaceful cofeeding groups may result from swamping where lower-ranking group members overrun dominants as a mob. In this scenario, the dominant individuals simply lose control. Fighting would be pointless and only make them lose feeding time and reduce their share while others are feeding. Studying feedings of free-ranging dogs, we show that aggression by dominants decreases with increasing feeding group size, which supports this alternative explanation and sheds new light on the emergence of cofeeding and social tolerance.

**Supplementary Information:**

The online version contains supplementary material available at 10.1007/s00265-025-03590-8.

## Introduction

Living and foraging in groups causes feeding competition between individuals. In theory, individuals should aim to compete for and monopolize food patches whenever economically feasible (Sterck et al. 1997). Competition can be high or low, and it can be of two forms, contest or scramble competition, with natural scenarios typically involving both components to varying degrees (van Schaik 1989; Sterck et al. 1997). Contest competition prevails whenever a valuable resource can be economically monopolized by one or more individuals against other individuals, typically because the resource occurs in a highly clumped patch (van Schaik 1989). Scramble competition prevails in all scenarios where a resource cannot be economically monopolized, e.g. when a resource occurs in small, distributed patches of equal quality or in a sufficiently large patch that serves all individuals (van Schaik 1989; Koenig 2002; Koenig and Borries 2006). Under scramble competition, fighting for a resource is pointless, and the only opportunity to increase one’s own share is through faster exploitation of the resource, which makes fighting actually a costly strategy as it reduces feeding time and efficiency and thus one’s share (Monaghan and Metcalfe 1985; van Schaik 1989; Estevez et al. 2007). Under contest competition, one or few individuals can monopolize the resource at the cost of other group members, hence the formation of a clearly defined dominance hierarchy gains benefits for dominant individuals through ensuring their priority of access to the resource (van Schaik 1989). In a pure contest scenario, the number of feeding individuals matches the number of individually defendable feeding patches (Altmann 1962; Koenig 2002; Koenig and Borries 2006). Cofeeding is absent, and all other, lower-ranking group members have to queue (= wait) for access to food, and all aggression is directed down the hierarchy (Preuschoft and van Schaik 2000). Since individuals are predicted to monopolize food patches whenever economically feasible, we will take pure contest competition as the “default” scenario in our study to outline and investigate scenarios that may facilitate cofeeding.

In practice, animals can be observed to peacefully cofeed on the same food patch, with particular large and dense cofeeding groups at large food patches (Sterck et al. 1997; Macdonald and Johnson 2015; Dale et al. 2017; DeTroy et al. 2022), Fig. 1). Three mutually non-exclusive hypotheses have been proposed to explain this behaviour. First, the “resource dispersion hypothesis” proposes that also under pure contest competition, peaceful cofeeding within a defendable area can occur on sufficiently rich food patches without costs to high-ranking individuals as long as their share is ensured (Macdonald 1983; Macdonald and Johnson 2015). Cofeeding will still follow a rank-dependent priority of access model since the high-ranking cofeeding individuals will only allow further lower-ranking individuals to join as long as their share is ensured (Berghänel et al. 2025). But if a food patch is rich enough to feed multiple individuals, then competition is virtually absent among these individuals. If cofeeding results solely from this mechanism, then we predict that access to food patches is strictly related to dominance rank, that aggression rate particularly by top-rankers increases with feeding group size relative to food patch richness, and aggression is directed down the hierarchy (Macdonald and Johnson 2015; see also Hanya 2009; Heesen et al. 2014; Richter et al. 2015; Rose and Soole 2020; Vogel and Janson 2007). Moreover, the special scenario of a particularly rich food patch relative to group size may allow all individuals to cofeed without any actual competition and rank effects (Hidalgo-Mihart et al. 2004; Macdonald and Johnson 2015).

**Fig. 1:**
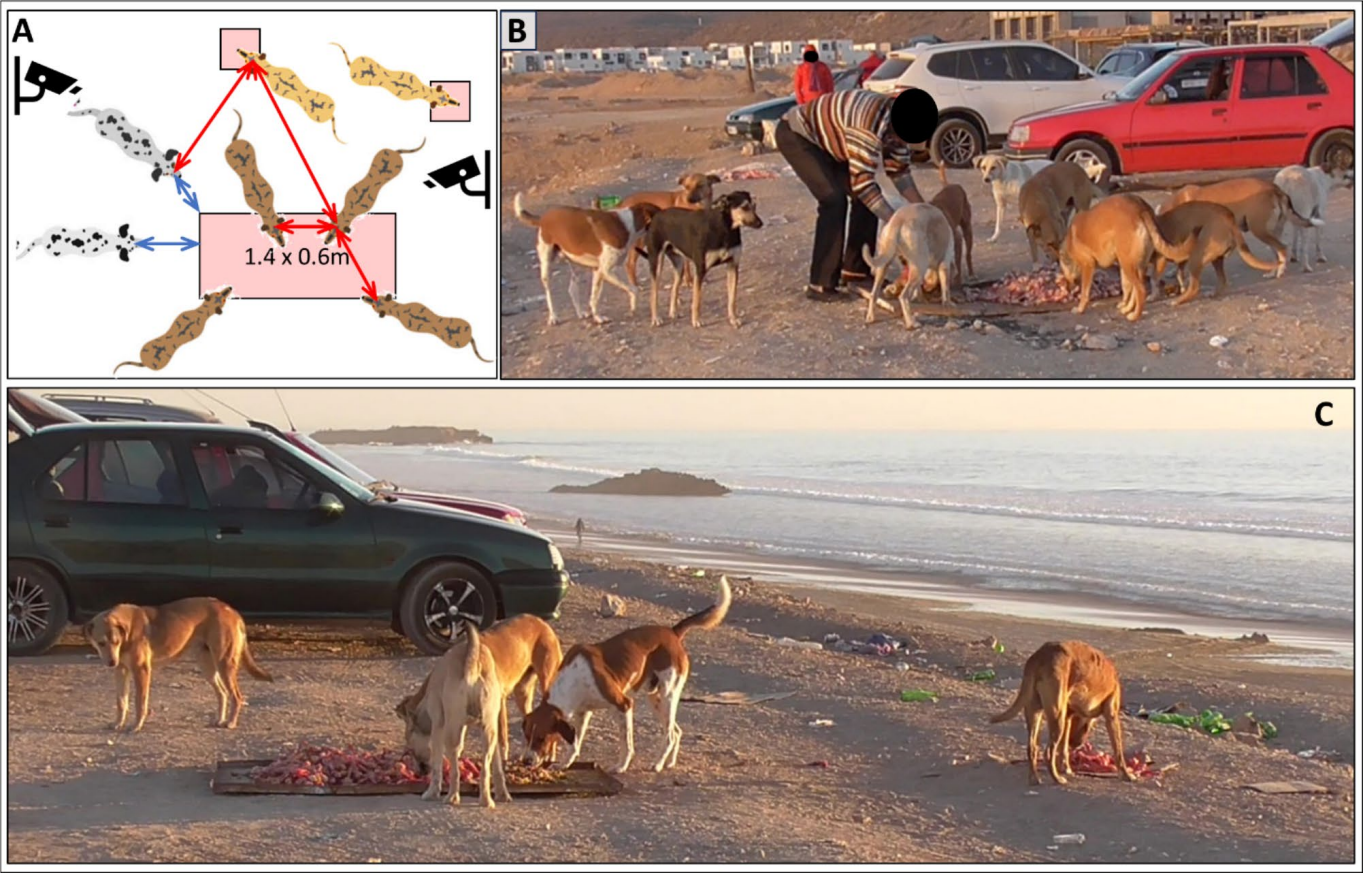
Test setup. Dogs from two neighbouring groups (distance ca. 900 m) were provided with slaughter waste by a local. The food was provided on a large pile on a 1.4 × 0.6 m metal platform which was not economically monopolizable and consumable by one but probably by two individuals (the “main food patch”), plus a varying number of up to four small high-quality food patches of ca. 40 × 40 cm that could each be easily monopolized and consumed by a single individual. (**A**) Schematic setup. Distances between individuals were recorded as head-head distances (red arrows) and distance to the main feeding patch as the shortest distance between head and patch border (blue arrows). Individuals were recorded as waiting (white dogs) or feeding (brown dogs), with further differentiation between feeding at the main food patch (dark brown) or not (light brown). (**B**, **C**) Test scenarios with waiting dogs and dogs feeding on the main food patch, and without (B) and with one (C) additional small food patch

Second, the “social tolerance hypothesis” predicts that under certain conditions, higher-ranking individuals may benefit from being socially tolerant and allowing lower-ranking individuals to cofeed (Sterck et al. 1997; DeTroy et al. 2022). Under prevailing contest competition, strict monopolization can be costly for dominant individuals if it causes disproportionate disadvantage to close kin, or to required collaboration partners which may then refrain from future collaboration or even leave the group (Sterck et al. 1997; Ostner and Schülke 2014; de Oliveira Terceiro et al. 2021; DeTroy et al. 2022). Under such conditions, it may be more beneficial for dominant individuals to not fully insist on their priority of access, but rather be socially tolerant towards lower-ranking individuals and allow them to co-feed, in particular if they are close kin or individuals with whom they have close social bonds (the “social tolerance hypothesis”; Sterck et al. 1997; Ostner and Schülke 2014; Elbroch et al. 2017; DeTroy et al. 2022). Hence if cofeeding is enabled by social tolerance, then access to food patches and likelihood of cofeeding will increase with social relationship strength and relatedness (Sterck et al. 1997; Staes et al. 2022). Rank effects are still predicted because social tolerance means concessions given by higher- towards lower-ranking individuals, hence cofeeding will cluster around top-ranking individuals (Sterck et al. 1997; DeTroy et al. 2022). In this scenario, aggressions will also be directed up the hierarchy (including counter-aggressions) due to the leverage and negotiation power by subordinates (Preuschoft and van Schaik 2000).

Third, the “swamping hypothesis” suggests that large groups can cause strong scramble competition by “swamping”, i.e., through high numbers of lower-ranking competitors that make an economic defence and monopolization of a clumped food patch unfeasible (Estevez et al. 2007). The gain of aggressive exclusion of single or few individuals decreases with increasing number of cofeeding competitors, whereas the costs of fighting in terms of a reduced share due to reduced feeding time and efficiency increase with an increasing scramble competition component. Hence aggression rates by the top-ranker(s) may still increase with the number of competitors in small groups due to prevailing contest competition, but should then decrease with further increasing feeding group size at the food patch due to prevailing scramble competition. Remaining monopolization attempts and thus aggression will still be strictly directed down the hierarchy. The “swamping hypothesis” was originally labelled the “social tolerance hypothesis” (Estevez et al. 2007), but for matter of accuracy (DeTroy et al. 2022) and clarity (i.e. to differentiate it from the social tolerance hypothesis above), we refer to it as the “swamping hypothesis” here. This hypothesis was mainly discussed for farm animals where decreasing aggression rates with increasing feeding group size were observed, but similar effects were also observed in other species like fish (Grant and Albright 2001; Andersen et al. 2004; Estevez et al. 2007).

Typically, these three mechanisms are difficult to differentiate because they result in very similar patterns of peaceful cofeeding. The aim of this study was to test and disentangle these different, mutually non-exclusive hypotheses by studying the feeding behaviour of a population of free-ranging dogs (FRDs; *Canis lupus familiaris*) in Morocco using observational and semi-experimental methods. FRDs provide a particularly promising model. Dogs are generalists with adaptations towards their anthropogenic niche and an omnivorous diet (Axelsson et al. 2013; Wang et al. 2013; Marshall-Pescini et al. 2017; Butler et al. 2018). FRDs live in an extraordinarily wide spectrum of social organization, ranging from solitary individuals through pairs and small groups to multi-male – multi-female groups of > 25 individuals, with high dispersal rates in both sexes and promiscuous mating throughout the year with some seasonality (Boitani and Ciucci 1995; Macdonald and Carr 1995; Pal et al. 1998; Chawla and Reece 2002; Bonanni and Cafazzo 2014; Cafazzo et al. 2014; Majumder and Bhadra 2015; Marshall-Pescini et al. 2017; Range and Marshall-Pescini 2022). They exhibit a clearly linear dominance hierarchy with formalized submission and dominance signals (Cafazzo et al. 2010; Bonanni et al. 2017). Although the point has not been directly investigated, a comparison of the limited data on the formation of territorial groups in FRDs suggest that such groups occur where food resources are predictable in space and collectively monopolizable, with group size increasing with abundance of food (Macdonald and Carr 1995; Dias et al. 2013; Bonanni and Cafazzo 2014; Krauze-Gryz and Gryz 2014; Range and Marshall-Pescini 2022).

FRDs emerged recently from a common ancestor with wolves (*Canis lupus*) 15–40 thousand years ago (Freedman et al. 2014; Skoglund et al. 2015; Wang et al. 2016) and remained genetically largely independent from artificially selected dog breeds (Pilot et al. 2015; Shannon et al. 2015). Wolves live in mainly family groups with cooperative breeding and strong mutual interdependence for collaborative hunting and territory and carcass defence, and as a consequence are a highly socially tolerant species with a clear dominance hierarchy but also high frequencies of cofeeding (Vucetich et al. 2004; Cubaynes et al. 2014; MacNulty et al. 2014; Cassidy et al. 2015; Range et al. 2015; Dale et al. 2017; Macdonald et al. 2019). Despite their different feeding ecology with preference for scavenging and rare pack hunting (Marshall-Pescini et al. 2017; Range and Marshall-Pescini 2022), dogs remained socially tolerant with high levels of cofeeding. In a dyadic feeding test on captive-housed mongrel dogs, individuals cofed peacefully from a 20 cm bowl in 97% of the test trials (Range et al. 2015 supplemental dataset; Dale et al. 2017), with increasing duration of peaceful cofeeding with increasing social relationship strength (Dale et al. 2017). Peaceful cofeeding also occurred when being provided with a high value medium-size food patch (leg of a deer) in a group setting, though in this setting the top-ranking individual also often monopolized the patch, with cofeeding occurring in 60% of the trials (Dale et al. 2017). In all scenarios there was a strong competition for first arrival at the patch (Dale et al. 2017), which indicates a strong scramble component in their feeding ecology.

**Fig. 2:**
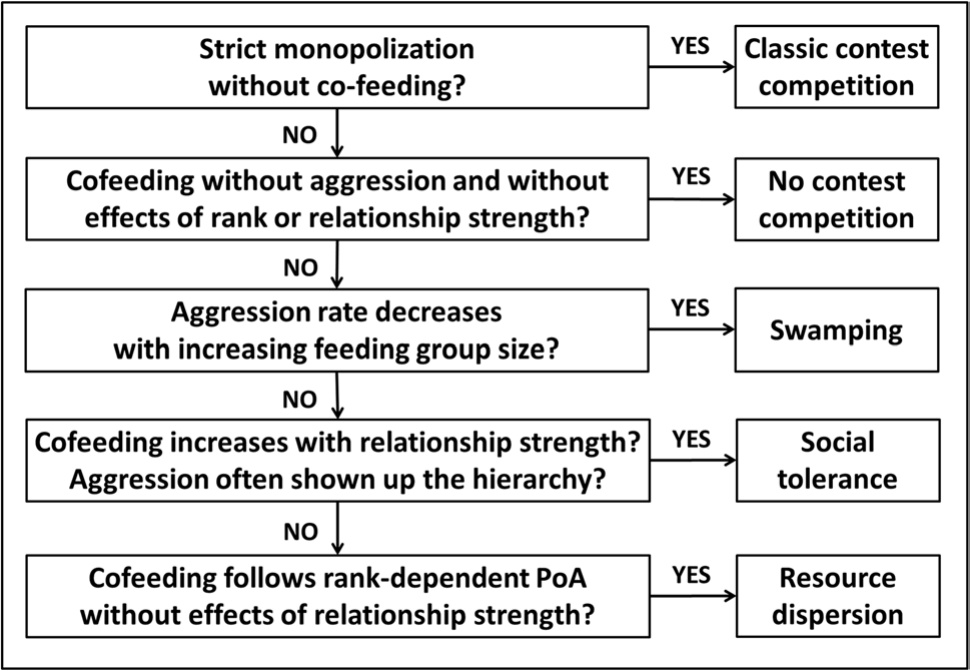
Predictions of the different hypotheses regarding behaviour at the large main food patch (Fig. 1). For a detailed outline see Table 1. Pure classic contest competition will be characterized by the absence of cofeeding within defendable range. Among the scenarios with cofeeding, social tolerance will be characterized by a positive effect of affiliative social relationship strength on (co-)feeding which is not predicted by the Resource Dispersion Hypothesis, whereas swamping is the only scenario where aggression rate is predicted to decrease with increasing feeding group size. The absence of any contest competition would be indicated by the absence of any aggression and rank effects. PoA: rank-dependent priority of access

In our study, we used an experimental setting to test distinctive predictions of the different hypotheses. Dogs were provided by locals with slaughter waste, which was delivered in one large high-quality food patch plus a varying number of small patches within 10 m around this main food patch (Fig. 1). The small patches could each be easily monopolized by a single individual, whereas the larger main food patch could hardly be economically monopolized by one but probably by two individuals (see Table 1 and Fig. 2 for detailed predictions).

**Table 1:**
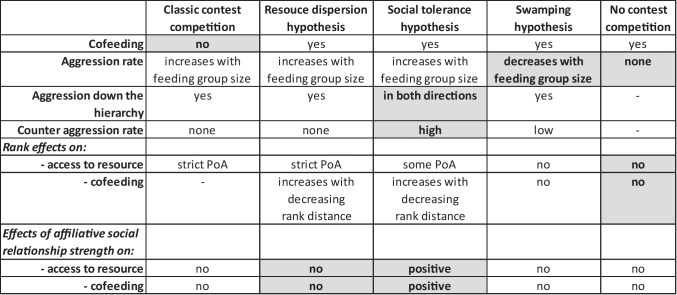
Predictions of the different hypotheses regarding behaviour at the large main food patch (Fig. 1; detailed outline of Fig. 2)

In bold with grey background: Main aspects for distinction. Pure classic contest competition will be characterized by the absence of cofeeding within defendable range. Among the scenarios with cofeeding, social tolerance will be characterized by a positive effect of affiliative social relationship strength on (co-)feeding which is not predicted by the Resource Dispersion Hypothesis, whereas swamping is the only scenario where aggression rate is predicted to decrease with increasing feeding group size. The absence of any contest competition would be indicated by the absence of any aggression and rank effects. PoA: rank-dependent priority of access

The small food patches allowed us to directly test our “default prediction”. We predicted that

- (pure) contest competition prevails around such highly monopolizable food patches.

If so, then

(i) small high-value food patches will always be monopolized by single individuals according to a rank-dependent priority of access.

If not, then

(ii) the alternative prediction will be true that social tolerance and cofeeding occurs even at small food patches. Thus, small high-value food patches will be shared with other group members according to the “social tolerance hypothesis”, and particularly between individuals that share a strong social relationship. This alternate prediction would then sufficiently explain cofeeding at the larger main food patch.

The large rich main food patch then allowed us to further address our main question:

b) Is access to food and to cofeeding at a large rich patch restricted by rank and social affiliative relationship strength, and what is the pattern of agonistic interactions?

This in turn allowed us to differentiate between the predictions of the three hypotheses at the main food patch:

(i) pure classic contest competition: aggression rate at the main food patch increases with feeding group size and is directed down the hierarchy; no peaceful cofeeding.
(ii) contest competition with cofeeding according to the Resource Dispersion Hypothesis: Rank-dependent access to and cofeeding at the main food patch, with no additional effect of affiliative social relationship strength. All aggression is directed down the hierarchy, and aggression rate by top-ranking individuals increases with feeding group size.
(iii) contest competition but with cofeeding according to social tolerance: The likelihood of both access to and cofeeding at the main food patch increases with social affiliative relationship strength, independent of parallel rank effects. Aggression rates either increase with feeding group size, are stable, or are generally absent, but do not decrease with increasing feeding group size. Aggression is also directed up the hierarchy.
(iv) cofeeding according to swamping and scramble competition: some level of contest competition prevails at low feeding group size, but with increasing feeding group size, scramble competition increases and thus aggression rate decreases.
(v) no contest competition: no aggression at the main food patch, and no rank or social relationship strength effects on access to and cofeeding at the main food patch.

## Methods

### Study population

The study was conducted on the Tamraght coast located near the city of Agadir in Morocco. The Tamraght coastal region is a hilly, semi-arid area located in the foothills of the Atlas Mountains and along the Atlantic Ocean. The climate varies little between seasons. This study site was founded in 2016 as part of the Domestication Lab unit of the Konrad Lorenz Institute of Ethology and encompasses 6 km of beach between the two villages of Taghazout and Tamraght. It was not possible to record data blind because our study involved animals in the field.

Several groups of FRDs live in this area and all FRDs in the Tamraght region are habituated to the presence of humans. All the FRDs of our population were free-living and could breed and move freely, and were not constrained or owned by humans. This makes them less exposed to artificial selection and more subject to natural selection pressures, which are comparable to those faced by wild canids (Pilot et al. 2016). They largely depend on humans for food, mostly from direct provisioning by local people but also by tourists and via restaurant and household leftovers. This provided us with the unique opportunity to conduct experimental feeding studies without ethical implications on their welfare and behaviour or disturbing their ecology. The individuals of our population form relatively stable groups around food sources, but group membership is fluid to some degree as individuals regularly change groups or visit multiple groups (personal observation). All FRDs of our study were individually recognized according to their morphology, coat colour, sex and age-class. Female reproductive status as well as general demographic data (e.g. births, deaths, migration) were recorded.

### Experimental setup

Experiments were carried out opportunistically from October 2017 to February 2018 by Martina Lazzaroni at a feeding site that was visited by two adjacent groups with 28 individuals in total (for a detailed description of the setup see Fig. 1). The two groups lived in two areas with high visibility and close to each other (900 m, with no other group in between). Individuals from both groups frequented both areas to some degree and visited the same feeding site located next to both areas, characterized by a medium abundance of food from direct human provisioning (butcher leftovers). We studied agonistic and affiliative interactions for all 28 individuals. However, our final subsample for analysis consisted only of the 17 individuals which were also present during the feeding sessions (see below).

### Feeding events

We recorded 15 complete feeding events in total on 15 different days, from which 8 events matched our inclusion criteria, i.e. a) events without mating competition context (no female in heat, excluded *N* = 2) and b) events where at least half of the present dogs showed any interest in the food (excluded *N* = 5). Food was distributed on a specific iron plate of about 1.4 m × 0.6 m in size which could hardly be monopolized by one but easily by two individuals, plus a varying number of small well-defined patches of about 0.4 m in diameter which could be easily monopolized by a single individual (Fig. 1). All food patches were at least 2 body lengths of ca. 0.8 m without tail (see below) apart from each other. Two synchronized cameras (Panasonic HC-V550 with FullHD and 50 × optical zoom) were used for video recordings, one to directly film the feeding and another to record all other behaviour ad libitum. Recordings started in the moment the food was placed on the plate, and stopped after the food was finished, or all individuals had stopped feeding (after up to 2 h). We analysed only the first 20 min of the records since individuals typically started losing interest in the food after this time. We analysed the behaviour of all individuals present in the feeding area at any of the included feeding events (*N* = 17).

### Behavioural observations

All-occurrence behavioural observations (ad libitum sampling, Altmann 1974) were conducted on the feeding videos and on non-feeding videos that were systematically recorded across the two areas during the study period. The non-feeding observations were conducted by one observer per area between 7am and 4 pm (26 observation days yielding 138.9 observation hours equally distributed across the two areas). These videos allowed us to analyse the effects of social affiliative and rank relationships shown in the absence of competition over food resources and how they influence the behaviour in the feeding competition context.

For both the feeding and the non-feeding observations, we recorded all agonistic and affiliative behaviours and the actor and the receiver of all interactions in Microsoft Excel (see Supplemental Table 4 for complete ethogram). Agonistic interactions included a) low aggression (non-contact aggression; staring, barking, baring teeth, raising hackles, jaw spar, snapping, pointing and lunging), b) high aggression (contact aggression; attacking, knocking down, pinning, biting, fighting and chasing), c) dominance displays (standing tall, standing over conspecific body, riding up, placing a paw on conspecific body, putting the head over conspecific back, and muzzle biting), and d) submissive behaviours (avert gaze, head dip, flattening ears, tail dip, crouching, fleeing, belly exposure, withdrawing, avoidance and whimpering). Affiliative interactions included approaching a conspecific within one body length, body contact of more than 10 s, grooming, bowing, playing, to lie, stand or approach friendly with tail wagging, short body rubbing, nose-to-nose contact and social sniff (all non-agonistic). All interactions were recorded as events, apart from body contact, grooming and social play for which we also recorded the duration. We further applied scan sampling every 5 min during the non-feeding observations (860 scans for each area). During these scans, we recorded all individuals present in the area, from which we calculated the proportion of scans that both individuals of a dyad were seen together in the same area (i.e. within a 200 m range) as an estimate of group membership and familiarity in our relatively fluid system (hereafter "familiarity").

To determine the actual monopolization of the food resources during the feeding sessions, we further applied scan sampling (Altmann 1974) at 1-min intervals. We recorded whether individuals were feeding or waiting (queuing) for access to food. Waiting was applied only if it was ensured that the respective individual began feeding later during the feeding sessions, e.g. after other individuals left the feeding patch (using the entire video time). To investigate co-feeding behaviour, we further recorded the dyadic head-to-head distances between all individuals present in the feeding area as well as the shortest distance between each individual and all feeding patch(es). To record these spatio-behavioural scan data, we recorded the spatial position for each food patch and ID and in case of ID also its behaviour in specific slides in Microsoft PowerPoint and then exported the coordinates in the slide (see Supplemental Fig. 1). The first feeding session was also coded by AB, yielding an interrater reliability of Cohen’s kappa = 0.953 for identification of individuals (with deviations only resulting from whether an individual was still within the observation range) and Cohen’s kappa = 0.689 for behaviour, which reflects substantial agreement according to (Landis and Koch 1977). Deviations in behavioural recording were mainly due to uncertainty in whether an individual was only resting or actively waiting for access to food.

### Behavioural analyses

We calculated a dominance hierarchy among the 28 individuals which were observed during the non-feeding observations (Supplemental Fig. 2). We used a winner-loser matrix based on the count of submissive behaviours during decided conflicts (unidirectional submission without counter-aggression) from the non-feeding observations, following (de Waal and Luttrell 1989; Bonanni et al. 2017). We calculated normalized David’s scores (DS) which provide a metric measure of dyadic dominance relationships (Gammell et al. 2003), with the highest score being held by the highest ranked individual and the lowest score by the lowest ranked individual. We tested the corrected Landau’s index of linearity h’ and the transitivity of the dominance hierarchy among the 28 individuals (de Vries 1995; Neumann et al. 2018) using R-package EloRating (Neumann et al. 2011). The statistical significance of the linearity index h’ was tested using a 2-step randomization test with 10 000 randomizations (de Vries 1995). We further computed the directional consistency index (DCI), which indicates the proportion of dyadic conflicts that were decided in the main direction within dyads, i.e. whether all were decided in the same direction or in both directions to varying degree (indicating an unsolved dyadic dominance relationship; van Hooff and Wensing 1987; for results see Supplemental Fig. 2). For each dyad, we calculated the absolute rank distance as the difference in DS, i.e. a measure of disparity between the respective dominance positions (de Waal 1991), and noted the top rank of each dyad as the DS of the dominant individual in each dyad (see below for rationale).

From the non-feeding observation data, we further calculated two separate measures of dyadic relationship strength between these 28 individuals, reflecting general familiarity and affiliative relationship strength, which both probably define different aspects of social relationship quality (group membership and social bonds). To separate these two aspects, we calculated familiarity (if two individuals were in the same area, see above) and affiliation rates corrected for familiarity (i.e., how often a dyad interacted affiliatively with each other if they were together in the same area). We then used these affiliation rates to calculate a dyadic “composite sociality index” (CSI) (Silk 2003; Silk et al. 2010). The CSI was constructed for each dyad as follows: (A/ma + B/mb + C/mc + D/md + E/me + F/mf + G/mg)/7."A"represented play behaviour count within a dyad, divided by the average (ma) of the play behaviour counts across all 136 dyads. The same calculation was used next for play duration (B), body contact count (C), duration of body contact (D), grooming count (E), duration of grooming (F) and other affiliative behaviours count (G). These values were summed together per dyad and then divided by the number of variables added together (= 7) to maintain a mean of 1 for the summed mean-scaled variables. The CSI measures the extent to which each dyad deviates in their sociality from the average dyad which by definition has a CSI of 1 (Silk 2003; Silk et al. 2010).

### Kinship—Reconstruction of genetic relatedness patterns within the study population

208 saliva samples were collected from 202 individuals from the study population (with six individuals sampled twice) using Performagene PG 100 saliva collection kits (DNA Genotek, Canada; for details of analyses see Supplemental methods). DNA samples were only available for 11 of the 17 individuals present in the feeding area at the feeding events, hence relatedness was only available for 40.4% of the dyads. We could therefore not investigate the role of kinship in our complex statistical models. However, we could analyse whether within the dyads with known relatedness, individuals were also seen to cofeed with lower-ranking unrelated individuals. For the purpose of this study, we defined all fourth-degree relatives and individuals related at more distant levels as “non-related individuals” following (Belisle and Chapais 2001; Chapais et al. 2001; Chapais and Berman 2004; for more information see also Supplemental Fig. 3, 4). Even though this is a simplification, the fourth-degree relatedness level is clearly distinct from first- and second-degree relatedness, and may be difficult to distinguish from the true lack of relatedness by the means available to animals, e.g. based on differences in individual scents (Silk 2002a; Kappeler and van Schaik 2006; Smith 2014).

### Statistical analyses

All statistical analyses were done with R version 4.3.0 (R Development Core Team 2020). All models were checked for model assumptions and binomial models for overdispersion using R-package DHARMa (Hartig 2024). If not stated otherwise, we run GAMM models (package mgcv (Wood 2015)) with cubic regression splines to account for potential non-linear effects in our predictor and control variables. For example, individual motivation to feed and monopolize may be high during the first minutes of a feeding event but then decline increasingly fast with increasing time. Similarly, rank-dependent priority of access does typically show a curvilinear relationship of access over rank (Dubuc et al. 2011; Sukmak et al. 2014; Higham et al. 2021). Applying linear models to such non-linear relationships can not only cause type 1 and type 2 errors but may even cause entirely artificial results (Berghänel et al. 2023). GAMMs build on established LMM algorithms (Bates et al. 2015) but allow for the estimation of curvilinear relationships (Wood 2015, 2017). To avoid overcomplexity and unpredicted wiggliness, we limited non-linearity to its minimum (k = 3) which constrains curvilinearity to single convex or concave relationships. For all models with less than 20 data points per variable we limited the models to linear effects, but still run GAMM models a) to yield comparable results and b) because the respective GLMM models (package lme4 (Bates et al. 2014)) often failed to converge whereas the “identical” GAMM versions converged successfully. Model comparisons were conducted with function compareML (package itsadug, van Rij et al. 2015), which we slightly adjusted in the source code to ensure Chi-Square tests and p-value calculation for all comparisons. All models were tested against the null model including the scan and the random effects only, and all models were significantly different from the null model if not stated otherwise.

All models followed the same basic structure. First, to account for repeated measures, all models included random effects for scan per feeding session (slope or random smooth in case of non-linear models Wieling 2018; Pedersen et al. 2019)). In addition, all models building on individual values per scan further included ID as random effect, and all models building on dyadic values per scan further included ID1, ID2 and the dyad as random effects.

Second, all models apart from Model 1 (which is a pure intercept model) included the following socioecological variables:

For the general competitive setting:

- Scan per feeding session to address changes in motivation to feed and thus to monopolize or cofeed.
- General group size around the feeding session to account for general competition level and number of generally available individuals which further relates to the maximum number of rank positions at the feeding session. Controlled for the number of additional feeding patches, this reflects group size relative to the number of available food patches.
- Number of additional small feeding patches (in addition to the main food patch) that may buffer competition by allowing individuals who would otherwise have to wait and queue to feed, and/or allowing individuals to avoid feeding competition at the main food patch. Due to its low variability (ranging from 0 to 4) this variable was handled as a linear term in all GAMMs.
- Feeding group size at the main food patch serving as a control variable in Model 2–4 and as the central predictor variable for aggression at the main food patch in Model 6.

For the social relationships:

- Social affiliative and rank relationships were included in all models as predictor variables.

We first investigated descriptively whether the small high-quality food patches were monopolized by single individuals or shared. For this we calculated the percentage of scans where a certain food patch was either fed on by one individual or cofed on by two or more individuals.

#### Model 1: General level of cofeeding

Across all individuals and food patches including the main food patch, we tested whether high-ranking individuals monopolize access to food resource within a defendable range (radius) of 0.8 m head distance (ca. one body length without tail) according to a rank-dependent priority of access. We predicted that the number of lower-ranking co-feeders should always be zero in case of complete monopolization by high-ranking individuals. We ran a one-sample approach with the number of lower-ranking co-feeders per individual and scan as response variable and random intercept effects of dog-ID and feeding session. In this one-sample approach, we compared our samples to a predicted mean of zero (similar to a one sample t-test) while at the same time also controlling for repeated measures through implemented random intercept effects. For this purpose we run a LMM (packages lme4 and lmerTest; Bates et al. 2014; Kuznetsova et al. 2020) with the respective random intercept effects only (i.e. no fixed effects). Since the term of interest was the intercept, we compared this model to an identical model but where the intercept was suppressed (set to zero). We ran this model across all individuals as well as separately for the highest-ranking feeding individual only.

As it turned out that co-feeding within a range of 0.8 m often involved many dogs (see Fig. 1B, Fig. 4 results section), we additionally considered a more conservative range of 0.4 m head distance which represents a very close co-feeding proximity.

#### Model 2: Probability of waiting vs feeding

We investigated which individuals had access to food, and under which conditions. We run a binomial GAMM with logit-link function on whether a certain individual was waiting or feeding at a certain scan (for behavioural definitions see above), i.e., comparing the white and brown dogs in schematic Fig. 1A. For the social relationships, we added individual DS as well as the average of all CSI values and the average of all familiarity values between the individual and all other feeding individuals. Aggression before the scan (i.e., during the minute preceding the scan) was not significant (*p* = 0.93) and thus removed from the model to allow inclusion of the first scan.

#### Model 3: Probability of feeding at the main food patch vs feeding away from the main food patch

Among the feeding individuals, we investigated which individuals had access to the main food patch and which individuals fed away from the main food patch, including individuals that fed on the additionally provided food patches but also the rare cases where individuals stole some food and thereby “created” their own food patch away from the main food patch. This model is identical to Model 2 but with the individual probability of feeding at the main food patch vs feeding away from the main food patch in a certain scan as response variable (i.e., comparing the dark and the light brown dogs in schematic Fig. 1A, with waiting and other dogs excluded). Aggression before the scan was not significant (*p* = 0.72) and thus removed from the model to allow inclusion of the first scan.

#### Model 4: Cofeeding behaviour

We investigated the conditions that increased the likelihood for dyadic cofeeding within 0.8 m, first in general among all feeding individuals (i.e. on all brown dogs in Fig. 1A) and second more specific at the main food patch only (i.e. on the dark brown dogs in Fig. 1A only). We run a binomial GAMM with logit-link function on whether a certain dyad was cofeeding within 0.8 m or not at a certain scan. For the social relationships, we added the dyadic CSI and familiarity as well as absolute dyadic rank distance (difference in DSs) and the top-rank in the dyad (highest DS) to the model. The top-rank was included because if rank effects play a role, then cofeeding will necessarily cluster around top-ranking individuals. This could drive potential rank distance effects since increasing rank distance necessarily involves increasing top-ranks, with the maximal rank distance necessarily involving the highest-ranking individual. To control for this potential confounding effect and also to assess potential clustering of cofeeding around top-ranking individuals directly, we additionally included the top-rank to the model. Aggression before the scan was not significant (*p* = 0.94) and thus removed from the model to allow inclusion of the first scan.

#### Model 5: Feeding group size at the main food patch

Before investigating how feeding group size affects aggression rate at the main food patch (Model 6), we first investigated what predicts the feeding group size at the main food patch, running a Gaussian GAMM on one feeding group size value per scan. For the social affiliative relationships, we added the average of all dyadic CSIs and the average of all dyadic familiarity values at the main food patch to the model. We further added the average ordinal rank at the main food patch to investigate whether feeding group size reflects priority of access to some degree, i.e., small feeding groups consist of top-ranking individuals and feeding groups increase by progressively lower-ranking individuals joining. We used the ordinal rank among all individuals present at the feeding session rather than DS because this better reflects the principle of priority of access (“queuing number”). The model controlled for the general competitive setting (e.g. general group size and number of additional small feeding patches, see above).

#### Model 6

Finally, we investigated how feeding group size affects aggression rate at the main food patch. For this, we run a Poisson GAMM on how feeding group size in a certain scan affects the number of aggressive (including dominance) behaviours during the following minute until the next scan. We chose Poisson distribution because of the high number of zeros in the count response variable. Since increasing aggression rate may vice versa also cause decreasing feeding group size, we added the number of aggressive behaviours during the preceding minute to the model to control for reversed causality, thereby providing time series (Granger) causality (Granger 1969; Dahlhaus and Eichler 2003). Due to the size of the iron plate marking the main food patch (ca. 1.4 * 0.6 m, diagonal ca. 1.5 m), two individuals can feed from the plate without being in cofeeding range, whereas a third individual cannot. We therefore added feeding group size as a continuous variable as well as a dichotomous variable differentiating between group sizes < = 2 and > = 3, which allows aggression rate to be low for one or two individuals, sharply increase at a group size of 3 and then continuously decrease with further increasing group size (for a similar approach see Behringer et al. 2022). For the social affiliative relationships, we added the average of all dyadic CSIs and the average of all dyadic familiarity values at the main food patch to the model. Due to the logic of rank-dependent priority of access, we primarily run the model on the number of aggressive behaviours shown by the two top-ranking individuals present at the main food patch. However, since the two highest-ranking individuals were responsible for only 54% of the aggressive behaviours shown across the feeding events, we rerun the same model again on all aggressive behaviours shown at the main feeding patch by all individuals.

## Results

### Monopolization at small food patches

**Fig. 3:**
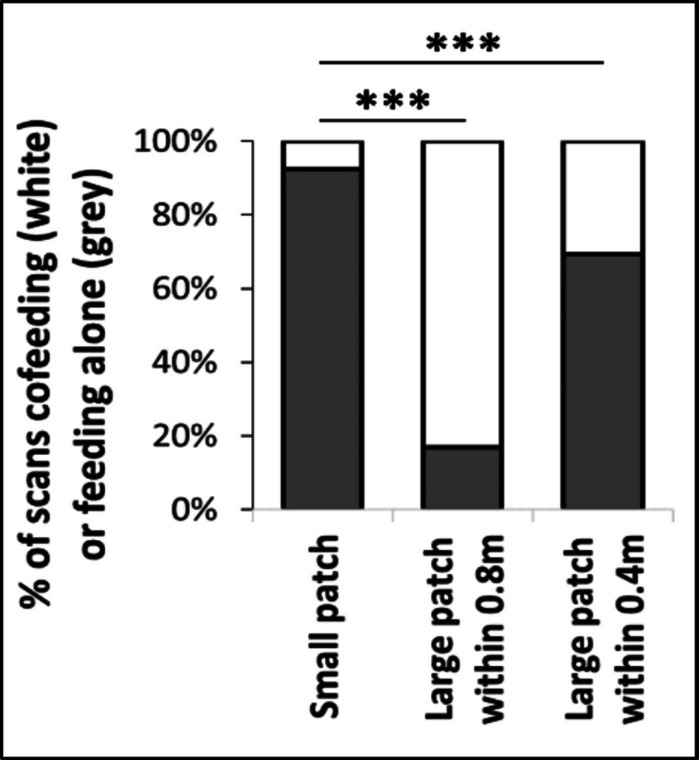
Proportion of scans where individuals fed alone or co-fed at the small and the large food patches. Proportions based on one value per food patch per scan. White: cofeeding, Grey: Single feeder. Left: small food patches, Middle: Large main food patch, cofeeding within 0.8 m head distance, Right: Large main food patch, cofeeding within 0.4 m head distance, which resembles more directly the cofeeding distance at the small food patches (see also Fig. 1 and Fig. 4 for more details). The number of scans is the same as for the large patch with cofeeding within 0.8 m (middle plot), just more scans are assigned to single feeders only instead of cofeeding due to the narrower definition of cofeeding

Single, small food patches were typically completely monopolized by one single individual and not shared. The small food patches were completely monopolized by one single individual at 92.5% of the scans whereas cofeeding with a second individual occurred during 7.5% of the scans (Fig. 3; no cofeeding of more than 2 individuals).

### Cofeeding at the large food patch

**Fig. 4:**
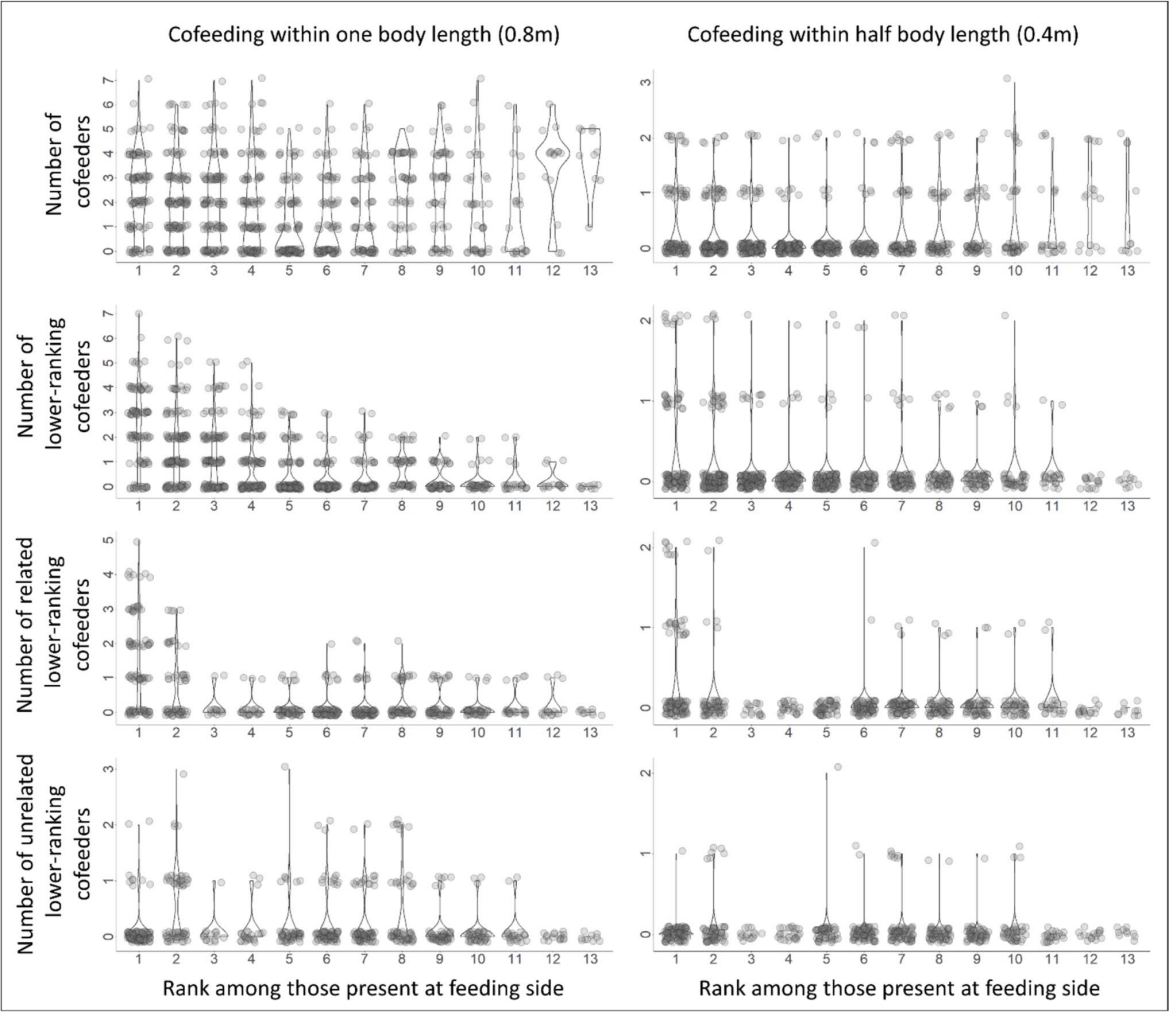
Number of co-feeders across scans and all feeding individuals, sorted by rank among the feeding individuals (Model 1). Descriptive plot to illustrate how often cofeeding occurred, and that it occurred by top-ranking and towards lower-ranking and unrelated individuals, and even within 0.4 m distance. The number of cofeeders was significantly different from zero in all cases, for the highest-ranking individual as well as across all individuals. Cofeeding distance: Measured as distance between heads. Rank: 1 = Highest ranking individual (among the individuals present at the feeding side, i.e. the same rank can be different individuals for different scans). Related and unrelated individuals: We defined fourth degree relatives (e.g. second degree cousins) or lower as unrelated and pairs related at the half-sibling level and above as related individuals. Relatedness was only available for 40% of the dyads, hence this figure does not show the complete number of related and unrelated individuals, but only that co-feeding with unrelated and lower-ranking individuals occurred. Violin plot with slightly scattered (jittered) data points for better visibility

In contrast, cofeeding occurred very frequently at the main food patch and often in very dense groups (Model 1), with up to 7 cofeeding individuals within 0.8 m around an individual and up to 3 cofeeding individuals within 0.4 m (Fig. 4; distances between heads; numbers of cofeeders significantly different from zero in all cases, for the highest-ranking individual as well as across all individuals). Cofeeding was not restricted to close kin, but occurred also with lower-ranking, unrelated individuals (Fig. 3). The percentage of scans with cofeeding was significantly higher at the large compared to the small food patches, also within a very close cofeeding range of 0.4 m head distance which resembles spatial conditions at the small food patches (Fig. 3; within 0.8 m: *X*^2^ = 210.0, *p* < 0.001, within 0.4 m: *X*^2^ = 32.5, *p* < 0.001).

**Fig. 5:**
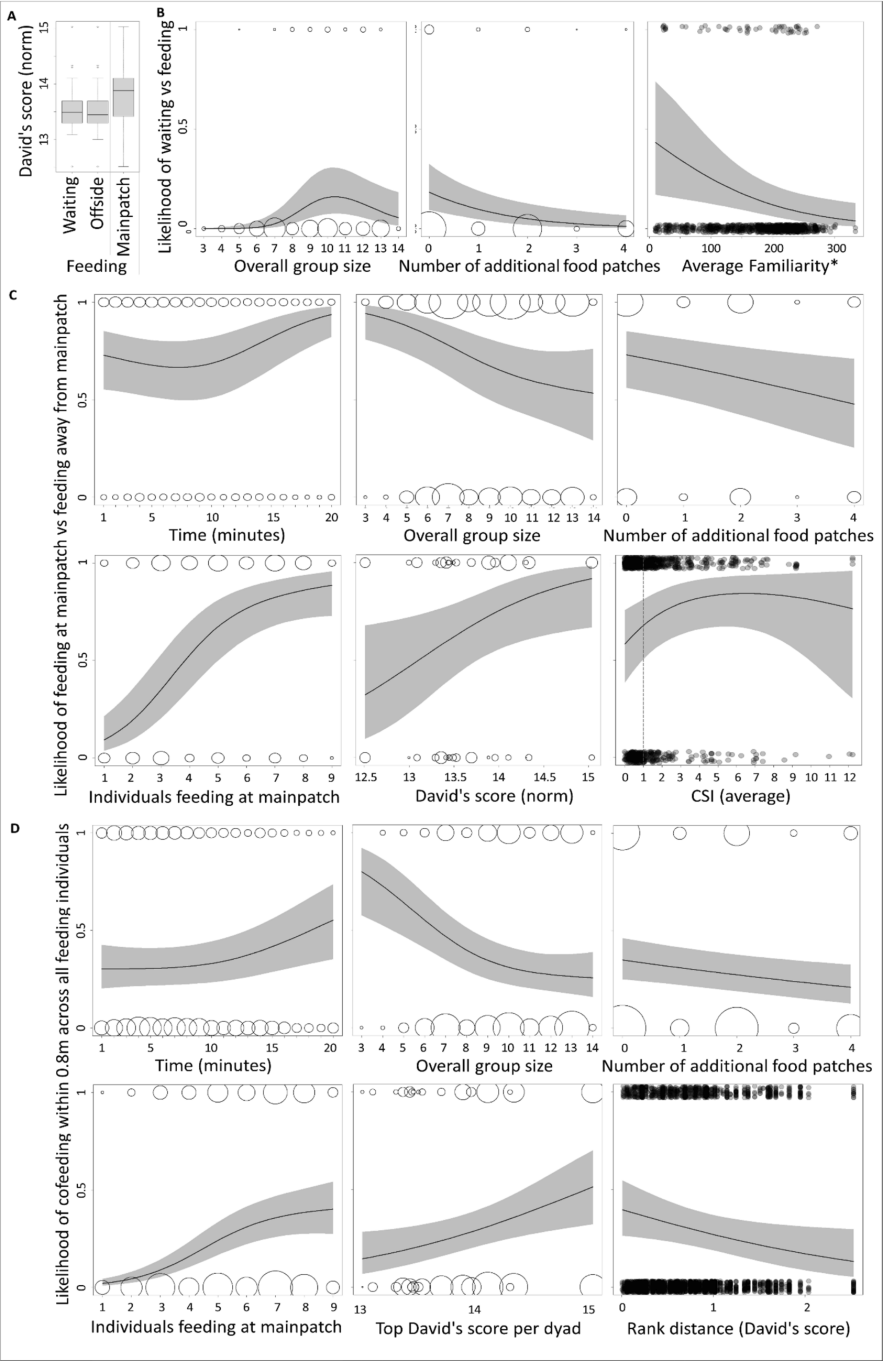
General feeding pattern at the feeding side (**B**-**D**). Size of open cricles: Number of cases with identical values (square-rooted). Filled data points: slightly scattered (jitter) for better visibility. Red line: CSI = 1, which marks the average relationship strength. Grey-shaded areas: 95% confidence intervals. **A** Difference in normedDS between waiting individuals, individuals feeding away from and individuals feeding at the man food patch (values per individual and scan). Higher DS were found at the main food patch whereas waiting individuals and those feeding at the “peripheral” food patches did not differ in their DS. Boxes indicate the inter quartile range (IQR), with the central line depicting the median and the whiskers extending to 1.5*IQR, and outliers. **B** Plots of the significant predictor variables from Model 2 (Table 2 left). The probability of an individual to wait at a certain scan increased with overall group size, decreased with the number of additional food patches, and increased with decreasing average familiarity with the feeding individuals. **C** Plots of the significant predictor variables from Model 3 (Table 2 middle). Most important, the probaility of an individual at a certain scan to feed at the main food patch increased with increasing rank (normed DS), whereas it was particularly low for individuals with a below-average CSI with the other feeding individuals. **D** Plots of the significant predictor variables from Model 4 (Table 2 right). Most important, the probability of a certain dyad to cofeed within 0.8 m distance at a certain scan increased with increasing top-rank involved in the dyad (i.e. cofeeding clustered around top-rankers) and increased with decreasing rank distance

The number of waiting individuals was generally low, and the individual likelihood of waiting (vs feeding; Model 2) increased with increasing overall group size and decreased with increasing number of additional food patches (Table 2 left, Fig. 5B). It further decreased with increasing familiarity, i.e. the average proportion of scans the individual was seen together with each of the (other) feeding individuals (Fig. 4B). It was not influenced by the average CSI with feeding individuals, nor by rank (but see Fig. 5A).

**Table 2:** Results of binomial GAMMs on the probability to wait (instead of feed, Model 2), to feed at the main food patch (compared to somewhere else, Model 3) and to cofeed within 0.8 m head distance (instead of feed, binomial GAMM, Model 4)

	Waiting vs feeding				Feeding at main patch or away				Dyadic co-feeding across all feeding individuals			
Linear terms	**Estimate**	**SE**	**z**	**p**	**Estimate**	**SE**	**z**	**p**	**Estimate**	**SE**	**z**	**p**
Intercept	− 3.25	0.84	− 3.86	< 0.001	1.24	0.36	3.48	< 0.001	− 0.90	0.23	− 3.94	< 0.001
Number of additional food patches	− 0.72	0.26	− 2.80	**0.005**	− 0.27	0.11	− 2.46	**0.014**	− 0.19	0.06	− 3.01	**0.003**
Smooth terms	**edf**	**Ref.df**	**Chi.sq**	**p**	**edf**	**Ref.df**	**Chi.sq**	**p**	**edf**	**Ref.df**	**Chi.sq**	**p**
Time (scan)	1.77	1.94	5.73	0.080	1.94	1.99	18.84	**< 0.001**	1.88	1.98	8.60	**0.009**
General group size	1.91	1.99	14.61	**< 0.001**	1.73	1.92	15.92	**0.002**	1.86	1.96	19.40	**< 0.001**
Feeding group size at main patch	1.00	1.00	1.53	0.22	1.89	1.99	71.64	**< 0.001**	1.97	2.00	91.72	**< 0.001**
CSI (average)	1.43	1.65	1.48	0.26	1.81	1.95	8.55	**0.023**	-	-	-	-
Familiarity (average)	1.00	1.00	6.06	**0.014**	1.00	1.00	1.23	0.27	-	-	-	-
DS	1.00	1.00	0.00	1.00	1.00	1.00	5.75	**0.017**	-	-	-	-
CSI (dyadic)	-	-	-	-	-	-	-	-	1.00	1.00	0.43	0.51
Familiarity (dyadic)	-	-	-	-	-	-	-	-	1.00	1.00	0.04	0.83
Absolute DS-distance (dyadic)	-	-	-	-	-	-	-	-	1.00	1.00	4.02	**0.045**
Top-DS in dyad	-	-	-	-	-	-	-	-	1.48	1.56	5.02	**0.029**
Random effects	**edf**	**Ref.df**	**Chi.sq**	**p**	**edf**	**Ref.df**	**Chi.sq**	**p**	**edf**	**Ref.df**	**Chi.sq**	**p**
Individual 1	10.80	19.00	40.20	< 0.001	15.63	19.00	125.24	< 0.001	13.18	19.00	122.36	< 0.001
Individual 2	-	-	-	-	-	-	-	-	12.89	19.00	159.47	< 0.001
Dyad	-	-	-	-	-	-	-	-	40.49	146.00	120.59	< 0.001
Scan per feeding session	5.68	7.00	30.23	< 0.001	4.62	7.00	18.46	0.025	6.21	22.00	32.63	0.001
	*N* = 1031, Deviance explained = 36.0%				*N* = 978, Deviance explained = 31.5%				*N* = 3018, Deviance explained = 18.2%			

Model 2 and 3: Individual values per scan. Model 4: Dyadic values per scan. *P*-values in bold: Significant predictor variables

Among the feeding individuals, the individual likelihood for an individual to feed at the main food patch (vs feeding offside; Model 3) increased with the number of individuals at the main food patch, and decreased with increasing overall group size as well as increasing number of additional food patches (which correspond to a higher proportion of individuals waiting or feeding away from the main patch; Table 2 middle, Fig. 5C). Beyond these general effects, the individual likelihood to feed at the main food patch increased with increasing rank and with increasing average CSI among the feeding individuals.

The likelihood of a dyad cofeeding within 0.8 m distance (Model 4) increased with increasing number of individuals at the main food patch, decreased with increasing overall group size and increasing number of additional food patches, and decreased with increasing rank distance (Table 2 right, Fig. 5C). It further increased with increasing top-rank in the dyad, hence cofeeding occurred mainly around high-ranking individuals, suggesting that they had some priority of access. Although the CSI predicted whether an individual had access to the main food patch in general (see above), it did not further influence whether a dyad was cofeeding specifically within 0.8 m or not.

**Fig. 6:**
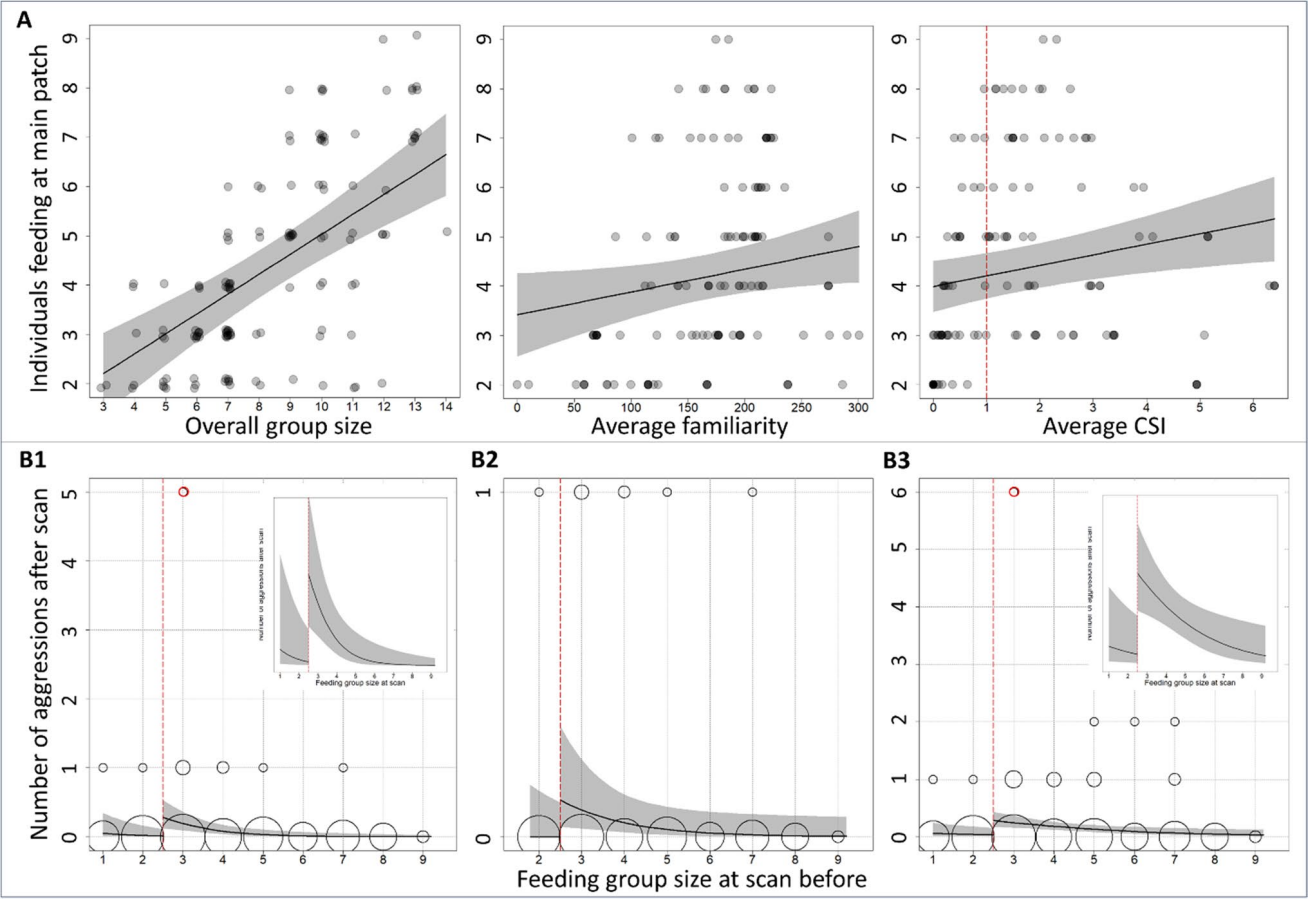
Predictors of the feeding group size and aggression rate at the main food patch. Grey-shaded areas: 95% confidence intervals. **A** Feeding group size at the main food patch increased with increasing overall group size at the feeding side as well as increasing average CSI and increasing average familiarity at the main food patch (Model 5, Table 3). Red line: CSI = 1, which marks the average relationship strength. Overall group size: data points slightly scattered (jittered) for better visibility. **B** The number of interaction bouts with aggressions and dominance behaviours at the main food patch first increased when a third individual joined and then decreased with further increasing feeding group size (Model 6). Small inlet figures: Zoom-in for better visibility. Red line: Theoretically, two individuals can feed in parallel at the main food patch without cofeeding, whereas a third individual could not. Red cricle: Potential outlier. Size of open cricles: Number of cases with identical values (square-rooted). **B1** The number of interaction bouts with aggressions and dominance behaviours shown by the two highest ranking individuals as in Table 4 left model (i.e. without the two sociality indices). **B2** The results from B1 also hold after including the two sociality indices and after removing the potential outlier (Table 4 right model). **B3** The results from B1 also hold if all aggressions and dominance signals from all individuals at the main food patch are considered (Supplemental Table 2 left)

Feeding group size at the main food patch (Model 5) increased with increasing average familiarity and increasing average CSI at the main food patch (Table 3, Fig. 6A). It further increased with increasing overall group size, but was not influenced by the number of additional feeding patches, indicating that these additional patches were not used to avoid tension and density at the main food patch, but only allowed lower-ranking individuals which would otherwise have to wait and queue to feed. Feeding group size at the main food patch was positively correlated with the average rank at the main food patch (rank assessed among all individuals present at the feeding session), showing that with increasing feeding group size, increasingly lower-ranking individuals join successively (see also Supplemental Fig. 5).

**Table 3:** Predictors of feeding group size at the main food patch (Model 5)

	Feeding group size at mainpatch			
Linear terms	**Estimate**	**SE**	**t**	**p**
Intercept	0.74	0.67	1.11	0.27
Number of additional food patches	− 0.21	0.14	− 1.48	0.14
Time (scan)	− 0.10	0.03	− 3.15	**0.002**
General group size	0.23	0.08	3.01	**0.003**
Average CSI at mainpatch	0.21	0.08	2.72	**0.007**
Average familiarity at mainpatch	0.00	0.00	2.12	**0.036**
Average rank at mainpatch	0.44	0.12	3.52	**0.001**
Aggression before	− 0.15	0.15	− 0.97	0.34
Random effect	**edf**	**Ref.df**	**F**	**p**
Scan per feeding session	5.17	7.00	4.70	< 0.001
	*N* = 134, R^2^ = 0.675, Dev. Expl. = 70.5%			

Gaussian GAMM on one value per scan. Aggression before: Number of aggression and dominance behaviours (interaction bouts) during the one minute between the preceding and the current scan. *P*-values in bold: Significant predictor variables

**Table 4:** Predictors of aggression rate by the two highest-ranking individuals at the main food patch (Model 6)

Aggression after by two highest ranking individuals at mainpatch												
Linear terms	**Estimate**	**SE**	**z**	**p**	**Estimate**	**SE**	**z**	**p**	**Estimate**	**SE**	**z**	**p**
Intercept	0.50	1.61	0.31	0.76	− 4.12	3.83	− 1.08	0.28	− 12.72	5.75	− 2.21	**0.027**
Feeding group size at mainpatch—Cut	3.54	1.37	2.59	**0.009**	8.35	4.18	2.00	**0.046**	9.95	4.78	2.08	**0.037**
Feeding group size at mainpatch—Cont	− 1.01	0.35	− 2.92	**0.003**	− 0.66	0.38	− 1.73	0.083	− 0.68	0.34	− 2.00	**0.046**
Time (scan)	− 0.19	0.07	− 2.60	**0.009**	− 0.15	0.10	− 1.50	0.13	− 0.12	0.11	− 1.11	0.27
Aggression before	0.79	0.31	2.53	**0.011**	1.80	0.65	2.10	**0.036**	2.55	1.04	2.44	**0.015**
Number of additional food patches	− 0.56	0.39	− 1.44	0.15	− 1.10	0.60	− 1.83	0.068	0.07	0.50	0.14	0.89
Average CSI at mainpatch	-	-	-	-	− 0.32	0.31	− 1.03	0.30	0.07	0.24	0.29	0.77
Average familiarity at mainpatch	-	-	-	-	− 0.01	0.01	− 0.97	0.33	0.02	0.01	1.62	0.11
**Random effect**	**edf**	**Ref.df**	**Chi.sq**	**p**	**edf**	**Ref.df**	**Chi.sq**	**p**	**edf**	**Ref.df**	**Chi.sq**	**p**
Scan per feeding session	1.29	7.00	1.55	0.30	2.28	7.00	3.31	0.19	0.18	7.00	0.20	0.34
	*N* = 144, Deviance explained = 37.4%				*N* = 129, Deviance explained = 47.8%				*N* = 128, Deviance explained = 47.7%			

Poisson GAMM on one value per scan. Aggression before: Number of aggression and dominance behaviours (interaction bouts) during the one minute between the preceding and the current scan. Aggression after: Number of aggression and dominance behaviours (interaction bouts) during the one minute between the current and the following scan. Right table: Without the potential outlier (Fig. 6B1 vs B2). *P*-values in bold: Significant predictor variables

### Feeding group size and aggression

**Fig. 7:**
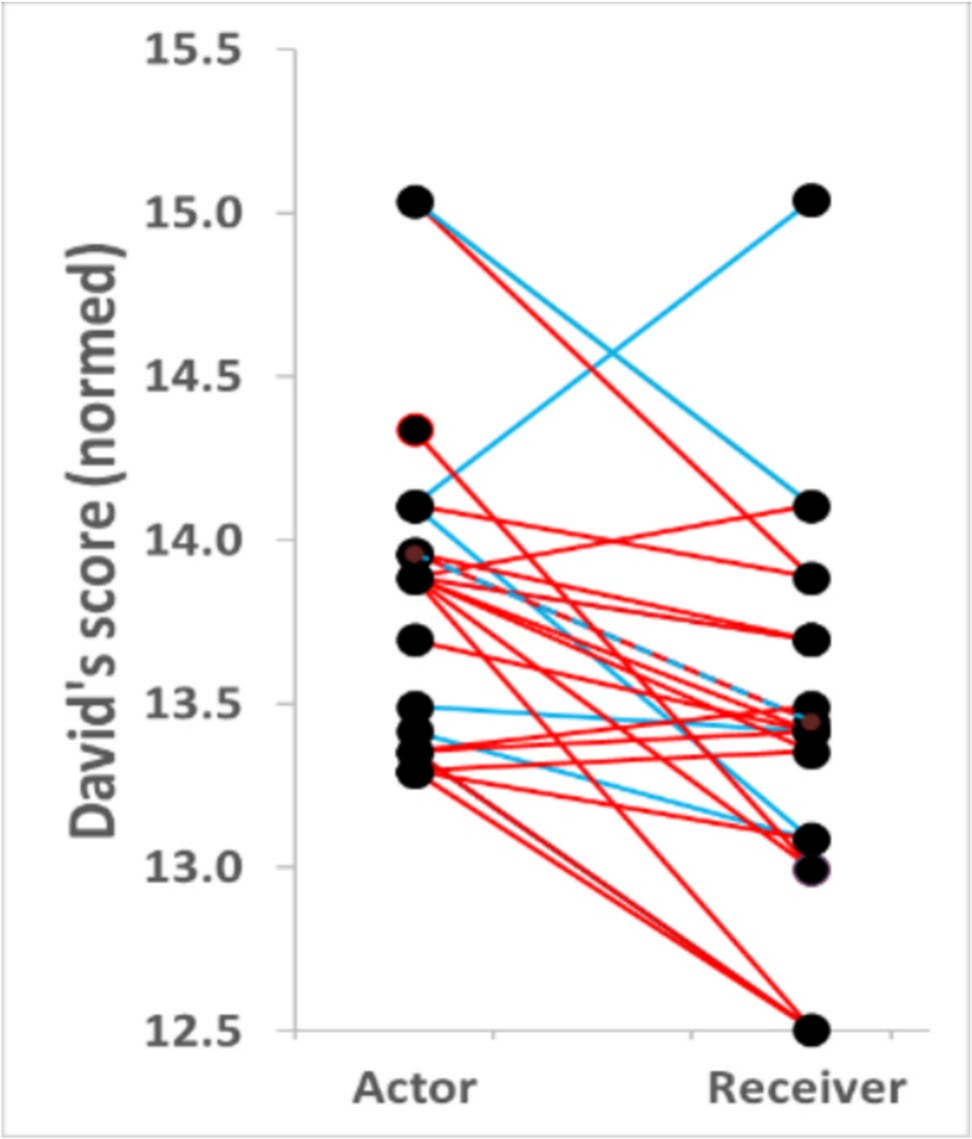
Direction of aggression. 84% of the aggressions and dominance displays were directed from higher- towards lower-ranking individuals (26 of 31, only aggressions: 21 of 25) and 16% directed up the hierarchy. Two of the 5 aggressions and dominance signals that were directed up the hierarchy were counter-aggressions (6.5% of all aggressions and dominance signals). Red: Aggressions, Blue: Dominance displays

Feeding group size at the main food patch at a certain time point (scan) predicted aggression rate by the two highest-ranking individuals during the following minute in the predicted way, even after controlling for the aggression rate during the preceding minute and thus reversed temporal causality (i.e., aggression reduces feeding group size; Table 4, Fig. 6B). Aggression rate was low if only one or two individuals were feeding at the main food patch, increased sharply when a third individual joined, and then decreased with further increasing feeding group size (Table 4 left, Fig. 6B1). 84% of aggressive behaviours were from higher- to lower-ranking individuals and 16% up the hierarchy (Fig. 7).

Feeding group size at the main food patch was positively related to our two sociality indices (see above, Table 3, Fig. 6A), which could mediate its negative relationship with aggression rate. Indeed, running the same model with either CSI or familiarity instead of feeding group size as predictor variable showed a negative effect of the average CSI or average familiarity on aggression rate at the main food patch (Supplemental Table 1), though these models had a lower model fit (model comparisons: all *p* < 0.003). Running a direct mediation analysis by adding feeding group size and our two sociality indices to the same model strongly inflated uncertainty, but the results suggest that the effect of feeding group size remained and was not or at least not completely mediated by relationship strength (Table 4 middle), and again the same model with the two sociality indices instead of feeding group size had a lower model fit (model comparison: *p* = 0.003). The found patterns remained similar after removing the potential outlier (Table 4 right, Fig. 6B2). Due to the high correlation between general group size and the feeding group size at the main food patch (r = 0.674, *p* < 0.001; Fig. 6A, Table 3), we could not meaningfully add general group size to the model. However, for all models, replacing feeding group size at the main food patch with general group size yielded significantly lower model fits (model comparison: all *p* < = 0.001).

Across the feeding events, the two highest-ranking individuals were responsible for 54% of the aggressive behaviours shown. Running the same models again on all aggressive behaviours shown at the main feeding patch yielded similar results (Fig. 6B3, Supplemental Table 2). However, in this case the results did not hold after removal of the potential outlier, yielding models that were not different from the null model.

### Potential role of kinship

**Fig. 8:**
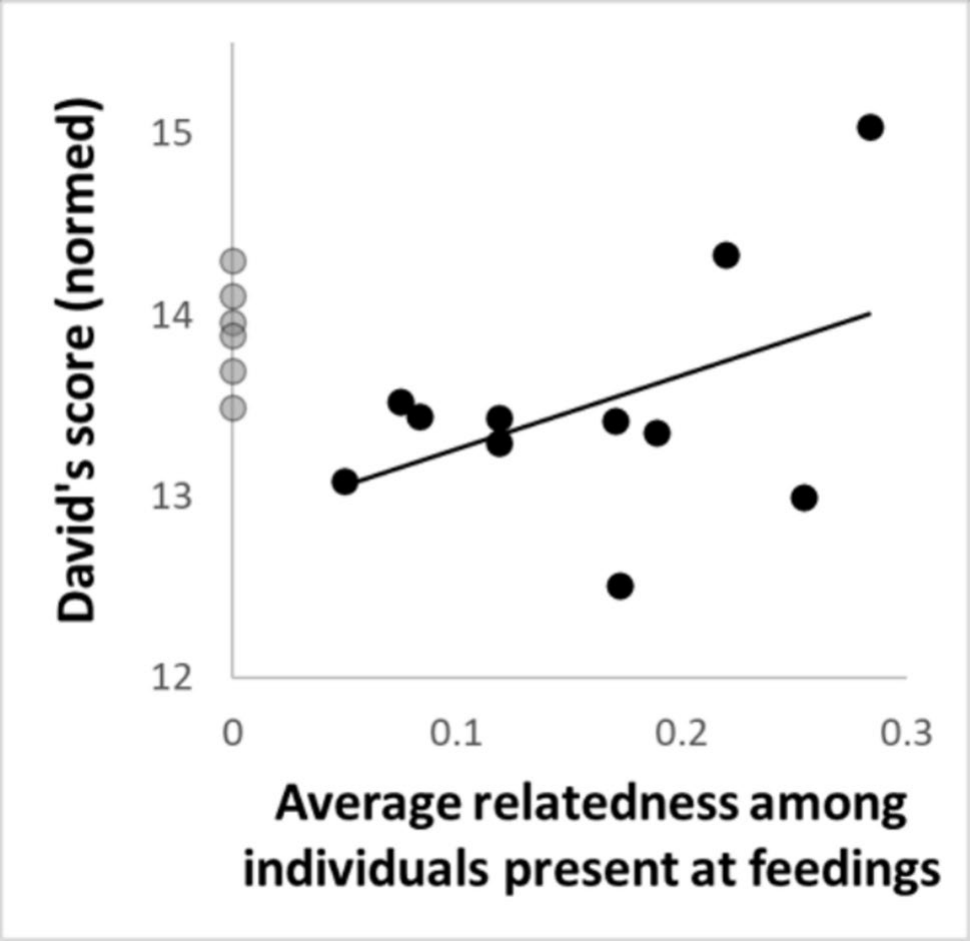
Average relatedness to other individuals and rank. Grey: normed David's score (DS) of individuals with unknown relatedness. The two top DSs were assigned to individuals with high average relatedness. However, missing DNA samples were biased towards individuals with rather high DSs (in grey), meaning that these individuals were not considered in the correlation nor were they considered in the calculation of the average relatedness values of the known individuals (in black)

Relatedness was only available for 40% of the dyads and could therefore not be implemented in our models, as this would have further increased model complexity while at the same time reducing sample size by more than 50%, excluding entire dyads and individuals. However, the sparse available data suggest that relatedness may not have been the main driver of our results. Across available dyads, relatedness was not correlated with familiarity (r = 0.046) or CSI (r = 0.167). Across scans, the average relatedness at the main food patch was not correlated with feeding group size (r = − 0.001) or number of aggressions after the scan (by the two top-ranking individuals: r = − 0.031; by all individuals: r = 0.017). However, among the 11 known individuals, two individuals with a high average relatedness with the other individuals also had a high dominance rank (r = 0.456, *p* = 0.16, *N* = 11; Fig. 8), and as a consequence, dyads with a high top rank also had high relatedness (across dyads; r = 0.427, *p* = 0.001, *N* = 55). Across dyads, also the proportion of scans a dyad was cofeeding within 0.8 m (only scans where both individuals were present) was positively correlated to relatedness (r = 0.301, *p* = 0.027, *N* = 55), which according to our main results could have been mediated by its correlation with the top rank in the dyad (Fig. 5D, Table 2). We therefore run a reduced model 4 on the likelihood of dyadic cofeeding within 0.8 m, including the rank effects and relatedness only (plus all control variables and random effects, see Supplemental Table 3 for details). Within this preliminary analysis, the likelihood of cofeeding was not related to relatedness (X^2^ = 0.53, *p* = 0.47) whereas top rank remained a significant predictor (X^2^ = 9.35, *p* = 0.005; effect of rank distance: X^2^ = 3.34, *p* = 0.068; for full model results see Supplemental Table 3).

## Discussion

Although small valuable food patches were almost always monopolized and consumed by single individuals, the dogs in our study usually cofeed in large and densely packed feeding groups at the larger food patch. These cofeeding groups provide deeper insight into whether cofeeding at the large food patch can be explained by the “Resource dispersion hypothesis” and thus pure contest competition at a superfluous resource patch, the “social tolerance hypothesis” and thus concessions by dominants to subordinates, and/or the “swamping hypothesis” and thus prevailing scramble competition caused by a large feeding group size. The cofeeding groups clustered around top-ranking individuals, and individual access to the feeding groups increased with increasing rank as well as increasing familiarity and affiliative relationship strength with the other feeding individuals. This indicates some remaining control over priority of access and a role for social tolerance in the formation of the cofeeding groups. Aggression rate at the large food patch was low for small feeding group sizes of one or two individuals, sharply increased if a third individual joined, and then decreased with further increasing feeding group size. Analysing time series causality showed that this effect was not driven by reversed causality, i.e. reduced feeding group sizes caused by increased aggression rates. The preliminary data on kinship effects suggest that they do not affect our other results and interpretations, though here more data are needed for final conclusions.

The rank effects found on the access to the main food patch suggest a certain level of contest competition and rank-dependent priority of access and reject the idea that cofeeding at the large food patch was the result of absent competition. However, individuals cofed largely peacefully in sometimes large and dense cofeeding groups at the main food patch, which also rejects pure contest competition. Independent of the rank effects, feeding group size and cofeeding density at the main food patch increased with increasing average affiliative relationship strength. These results indicate a strong role for social tolerance (DeTroy et al. 2022; Staes et al. 2022) and suggest that cofeeding at the large rich patch could not or at least not completely be explained by the neutral aggregation process proposed by the Resource Dispersion Hypothesis (Macdonald 1983; Macdonald and Johnson 2015). Finally, our results suggest that cofeeding was not only facilitated by social tolerance but at least in part also due to scramble competition and swamping effects (Estevez et al. 2007). At small feeding group sizes of up to 3 individuals, aggression rate increased with increasing group size, indicating prevailing contest competition in combination with social tolerance. However, further increasing feeding group size led to decreasing aggression rate even after controlling for reverse causality, suggesting prevailing and increasing scramble competition (Estevez et al. 2007). Preceding aggression did not reduce feeding group size in our analyses, indicating that aggression was on average not successful in excluding competitors.

The negative effect of feeding group size on aggression rate was not, or at least not completely, explained by the concomitant increase in affiliative relationship strength. However, here our results must be interpreted with some caution. Mediation analysis requires that the predictor variables are estimated with similar accuracy, but the number of feeding individuals at the main food patch was likely recorded with higher accuracy than affiliative relationship strength, meaning that feeding group size may just provide a more precise estimate of average social relationship strength than the average CSI. Hence, we cannot completely rule out that the reduced aggression rate in larger feeding groups was due to higher average affiliative relationship strength and thus directly linked to higher social tolerance rather than increased group size.

In any case, social tolerance likely acted as a starting point for the swamping, might it be through general loss of control or through social group dynamics, as e.g. allowing access to a friend may also open the door for their friends and the friends of their friends. Social tolerance may have enabled cofeeding between individuals close in rank and/or with strong affiliative relationships, with lower-ranking individuals then joining. This interpretation would be in line with previous results on captive dogs and wolves. Like in our study, cofeeding was positively related to affiliative relationship strength in these dogs and wolves, and food monopolization and cofeeding were strongly linked to dominance rank in dogs but not in wolves, with dominant individuals often monopolizing the food (Range et al. 2015; Dale et al. 2017) (see also Bonanni et al. 2017 for high social tolerance in free-ranging dogs in Rome, Italy). Provided with a small to medium sized valuable, monopolizable food patch, these dogs showed higher levels of social tolerance and cofeeding in a dyadic than in a group setting where the risk of swamping and scramble competition with many individuals would be higher (Range et al. 2015; Dale et al. 2017). When captive wolves were provided with a relatively large food patch (entire deer carcass) in a group setting, they often cofed in dense groups but were also often able to monopolize the entire carcass and exclusively feed on it, which supports the idea that cofeeding and swamping at such food patches requires some level of social tolerance. With this regard, it is however important to acknowledge that in our study, the large food patch could not be economically defended by a single feeding individual but easily by two, a notion that was supported by our results and specifically the low aggression rates in feeding groups of two and the rise in aggression rate if a third individual joined. Although this pattern and the effects of rank and affiliative relationship strength strongly suggest a role for contest competition and social tolerance, we cannot completely rule out that the required coordinated defence by two individuals while feeding required too much coordination for the dogs, leading to some loss of control and facilitating swamping directly.

However, the positive relationship between affiliative relationship strength and access to and feeding group size at the main food patch suggests a high level of social tolerance in our dog population. In line with this interpretation, aggression and dominance signals were mainly directed from higher- to lower-ranking individuals but were also exhibited up the hierarchy, as predicted for social tolerance and also previously shown for captive and free-ranging dogs (Preuschoft and van Schaik 2000; Cafazzo et al. 2010; van der Borg et al. 2015; Bonanni et al. 2017). However, the percentage of aggressions shown up the hierarchy may be affected by unknown relationships and thus uncertainty of dominance relationships. Counter aggression as an unaffected behaviour was however comparably rare and rather matched values reported for despotic-intolerant species (for a comparison with tolerant and intolerant primate species see e.g. Silk 2002b; Cooper and Bernstein 2008; Richter et al. 2009; Berghänel et al. 2011; Balasubramaniam et al. 2012). Aggression rate was generally very low though and of low intensity during feeding, allowing also multiple unrelated individuals to cofeed within very close head distances of less than 40 cm, which in itself provides strong proof of high cofeeding tolerance. Under such conditions, negotiations and counter aggressions may just not be as relevant, and aggressions and dominance signals from dominant individuals may just be taken as a communication of serious motivation and insistence on their priority of access.

Interesting insights into swamping and the ability of top-rankers to adjust their behaviour accordingly comes from other species. Swamping likely plays an important role in interspecies encounters at a common food resource, might it be wasps at a breakfast table or carcass defence by e.g. wolves, African wild dogs (*Lycaon pictus*) and lions (*Panthera leo*) against ravens (*Corvus corax*), hyenas (*Crocuta crocuta*) or vultures. Observations suggest that ravens and hyenas use swamping to gain access to carcasses (Vucetich et al. 2004; Lehmann et al. 2017), but to our knowledge no study investigated this explicitly. Turkey vultures (*Cathartes aura*) aggregate in roosts and use local enhancement and roost-centred food information transfer to aggregate at carrions in groups of up to 12 individuals (Prior and Weatherhead 1991). However, at carrions, they exhibit strong contest competition among each other without social cofeeding tolerance, avoiding spatial cofeeding and showing strong rank-dependent priority of access, with increasing aggression rate with increasing group size. This lack of cofeeding tolerance may prevent swamping, and vultures seem to never effectively swamp superior competitors like coyotes, cats or hawks (Prior and Weatherhead 1991). However, interspecies interactions are typically also characterized by zero social tolerance between the species, and the high risk of injury and lethal aggression may negate the potential benefits of a swamping strategy; hence swamping may generally be more likely in within- than between-species competition.

Intraspecific evidence is difficult to evaluate because swamping may often be difficult to distinguish from social tolerance, including in feeding groups that loosely aggregate and tolerate each other as anti-predator tactic (Elgar 1986). The intraspecific competition of vultures at carrions mentioned above suggests that even if theoretically possible, swamping may not always be the most economic strategy, or may rely on some level of social tolerance particularly in heavily armed species.

Intriguingly, in groups of rather solitary living wild brown hares (*Lepus europaeus*) that fed at one single clumped patch, aggression rates by dominant individuals increased with feeding group size, with the consequence that for feeding group sizes larger than three, subordinates spent the same or even a higher proportion of time feeding per individual than the dominants (Monaghan and Metcalfe 1985). Hence, swamping can also be feasible without social tolerance under certain conditions. Moreover, this example illustrates how blindly pursuing monopolization under such conditions can lead to disadvantages and maladaptive outcomes, but also that the behavioural flexibility required for an adaptive change in behaviour by dominant individuals seem to require evolutionary exposure and selection. This is further supported by evidence from different gregarious fish species where in contrast to the solitary brown hares, individuals were able to adjust their behaviour flexibly. Dominant individuals aimed to monopolize a clumped rich food patch at small group sizes but stopped doing so and switched to scramble competition at larger group sizes (Ruzzante and Doyle 1993; Syarifuddin and Kramer 1996; Grant and Albright 2001). Aggression rates decreased with increasing group size, and in large groups, socially indifferent and less aggressive fish gained higher food intake and faster growth than individuals that still contested for food (Ruzzante and Doyle 1991, 1993; Syarifuddin and Kramer 1996; Grant and Albright 2001).

Free-ranging dogs show an enormous flexibility in their social organization, ranging from solitary to temporal aggregations around food patches to large stable groups, with this variation being probably linked to variation in the amount and distribution of food (Bonanni and Cafazzo 2014; reviewed in Range and Marshall-Pescini 2022). Especially if food occurs in large and highly predictable patches that allow for group defence and the saturation of multiple individuals, large stable groups may form. Indeed the largest reported stable group of 27 free-ranging dogs was observed in the outcasts of Rome living under very similar conditions as our study group, namely daily provisioning with piles of slaughterhouse remains on defined feeding places (Bonanni and Cafazzo 2014). Here, our study contributes insight into the mechanisms that allow to overcome monopolization of such food patches by single or few top-ranking individuals and to provide access for all group members of such large groups. It shows particularly the flexibility of dominant individuals to adjust their behaviour to the actual competitive circumstances at the food patch.

Our results may encourage a discussion about to what degree social tolerance does not only mirror scramble competition in its effects but may often cause scramble competition. Our study suggests that scramble competition becomes increasingly important with increasing cofeeding group size. But irrespective of cofeeding group size, social tolerance may often drive subordinate and, as a consequence, also dominant individuals to apply scramble competition tactics. Socially tolerant pairs of semi-captive chimpanzees (*Pan troglodytes*) increased their intake rate during cofeeding compared to when they were feeding alone, or when they were cofeeding but on pre-assigned food portions (Koomen and Herrmann 2018). Highly socially tolerant wild common marmosets (*Callithrix jacchus*) regularly cofed at a single clumped food patch (63% of visits), but seemed to use scramble competition strategies (first arrival) to maximize their share (De la Fuente et al. 2019). In long-tailed macaques (*Macaca fascicularis*), both dominant and socially tolerated individuals used scramble competition tactics to maximise their share, and particularly subordinates did so more at large than small food patches (Dubuc and Chapais 2007). Anecdotally, cofeeding captive wolves and dogs showed strong behavioural scramble competition in both the dyadic and the group feeding setting (Dale et al. 2017 main text and supplemental videos). It might be intriguing to investigate this aspect in more species and under different conditions (e.g. general cofeeding vs equal sharing of collaboration gain) and to differentiate between social tolerance that just shifts competition from contest to scramble competition (i.e., from direct to indirect competition), and social tolerance that truly eliminates competition. Indeed, supressing scramble competition tactics may require strong self control and even behavioural rules and conventions around food patches also in humans. In contrast to"ecologically enforced"scramble competition, the initiation of such scramble competition via social tolerance is still under concession control by dominants and may also not apply to all individuals but be granted to selected individuals.

Our results are in line with several basic socioecological predictions (van Schaik 1989; Sterck et al. 1997; Koenig 2002; Koenig and Borries 2006; Clutton-Brock and Janson 2012). Even though individuals exhibited strong contest competition and almost always monopolized small valuable food patches, they very often cofed at large rich food patches (van Schaik 1989; Macdonald and Johnson 2015). The individual likelihood of exclusion from feeding depended on the relationship between group size and the number of feeding sites, and independently also on general familiarity, suggesting that some kind of ingroup-outgroup effects act also in our system of rather fluid rather than categorical group membership. Additional food patches were used by lower-ranking individuals to avoid competition at the large main food patch. However, additional food patches did not influence and were not directly used to escape competition at the large main food patch, but allowed lower-ranking individuals to feed that otherwise would have been excluded from feeding, as also found in other wild species (Vogel and Janson 2007; Hanya 2009; Heesen et al. 2014). The main conclusion of this study is, however, that cofeeding may not always indicate social tolerance but can also be due to swamping, where the presence of many individuals overwhelms the ability to monopolize resources, leading to scramble competition.

The results of our study may be of reduced generalizability for the following reasons. First, our results are based on a small sample, with only few feeding tests applied to the same dogs. Applying similar tests to multiple groups under different socioecological conditions would allow to address the aspects in more detail, including rank, kinship and social relationship effects and the role of between-group competition. This could also include different settings and species where access to food benefits from cooperation, as might be the case for group-hunting or for opening and defending large carcases (Creel 2001; MacNulty et al. 2014; Lehmann et al. 2017). Furthermore, since our study population was provisioned with food by locals on a regular basis, it probably faced lower competition for food than wild animals. This may influence our results in multiple ways. Social tolerance reflects concessions by dominants who are otherwise able to monopolize the resource, with the motivation to monopolize increasing and social tolerance decreasing depending on the dominant’s needs and hunger level (Preuschoft and van Schaik 2000). Similarly, the motivation and investment to defend a resource patch against crowds of competitors will depend on the value of the patch for the individual, which decreases with increasing general food availability and thus decreasing overall food competition. Whether and how a higher motivation would enable them to prevent swamping remains an open question. It will therefore be important to validate our results in wild animals and by comparing multiple socioecological conditions.

## Supplementary Information

Below is the link to the electronic supplementary material.

Supplementary file1 (DOCX 489 KB)

## Data Availability

The source data and R-code are permanently stored and available at Phaidra at 10.34876/y9k2-et75 and 10.34876/5yw9-ck82.
