# Supplementary material for: Cofeeding at rich clumped food patches in free-ranging dogs: social tolerance or scramble competition?
Source: Behav Ecol Sociobiol. 2025 Apr 14;79(4):51. doi: 10.1007/s00265-025-03590-8 (PMC11996968; doi:10.1007/s00265-025-03590-8)
Supplement: Supplementary file 1 — Supplementary file1 (DOCX 489 KB) [file 265_2025_3590_MOESM1_ESM.docx]

**Supplemental Material**

**Cofeeding at rich clumped food patches in free-ranging dogs: Social tolerance or scramble competition?**

*Behavioural ecology and sociobiology*

Andreas Berghänel^1#^, Martina Lazzaroni^2^, Malgorzata Ferenc^1^, Malgorzata Pilot^3^, Ikhlass el Berbri^4^, Sarah Marshall-Pescini^1^*, Friederike Range^1^*

1 Domestication Lab, Konrad Lorenz Institute of Ethology, University of Veterinary Medicine Vienna, Vienna, Austria

2 Department of Chemistry, Life Science and Environmental Sustainability, University of Parma, Italy

3 Faculty of Biology, University of Gdańsk, Gdańsk, Poland

4 Department of Pathology and Veterinary Public Health, Agronomic and Veterinary Institute Hassan II, Rabat, Morocco

* Authors contributed equally

# Corresponding author: [Andreas.Berghaenel@vetmeduni.ac.at](mailto:Andreas.Berghaenel@vetmeduni.ac.at)

*Supplemental methods*

Kinship - Reconstruction of genetic relatedness patterns within the study population

208 saliva samples were collected from 202 individuals from the study population (with six individuals sampled twice) using Performagene PG 100 saliva collection kits (DNA Genotek, Canada). Among these individuals, there were 11 individuals subject to the feeding experiments carried out in this study. DNA extraction was carried out according to the instructions of the manufacturer, with the use of PG-AC4 reagent (DNA Genotek, Canada) to remove impurities from the solution. The samples were genotyped at 163 594 autosomal SNPs (single nucleotide polymorphisms), using Axiom Canine HD Genotyping Array (Thermo Scientific). We used Plink1.9 software (Chang et al. 2015) for data filtering and relatedness analysis. We removed SNPs with a genotyping rate below 10% and those with a minor allele frequency below 5%, as well as those in strong linkage disequilibrium (with r^2^>0.1 within 50 consecutive SNPs, shifted and recalculated every 10 SNPs). After the filtering, we obtained a dataset of 20 377 SNPs with 98% genotyping rate, which we used to assess relatedness among the sampled individuals. Relatedness was estimated based on pair-wise identity by descent (IBD) coefficient PI_HAT and IBS coefficient DST, using --genome function in Plink. We excluded two samples with low genotyping rate, but both of them originated from individuals sampled twice, so this has not led to the reduction of the number of genotyped individuals studied.

We carried out the data analysis so as to maximize the number of samples included and therefore initially we did not remove any sample independent of the percentage of missing data. However, after the initial analysis, we removed two samples which produced spurious results. The PI_HAT coefficient ranged from 0 to 0.721 in the entire sample population, with an average of 0.071 (SD 0.098; Supplemental figure 2), while DST ranged from 0.612 to 0.887, with an average of 0.691 (SD 0.024). This excluded four pairs of samples originating from the same individuals, for which the PI_HAT coefficient ranged from 0.952 to 0.999, and DST from 0.981 to 0.999. This last result confirms the accuracy of individual identification based on visible morphological features we applied in this study and shows that we were able to replicate the individual SNP genotype with high, although not perfect, accuracy.

In further analyses, we relied on the PI_HAT coefficient, which showed values highly consistent with expectations for known relationships (mother-offspring pairs and maternal siblings). The distribution of PI_HAT values for individuals participating in the feeding experiments shows three clusters of individuals (Supplemental figure 3): (1) first-degree relatives (at the mother-offspring and the full-sibling level; expected PI_HAT value of 0.5), (2) second-degree relatives (at the half-sibling level, expected PI_HAT value of 0.25), and (3) from fourth-degree relatives (at the second-level cousins level, expected PI_HAT value of 0.0625) to non-related individuals (expected PI_HAT of 0). The absence of third degree relatives among these dogs was likely incidental, given that they were present in the entire dataset of 202 individuals (Supplemental figure 2).

*Supplemental Figures*

*
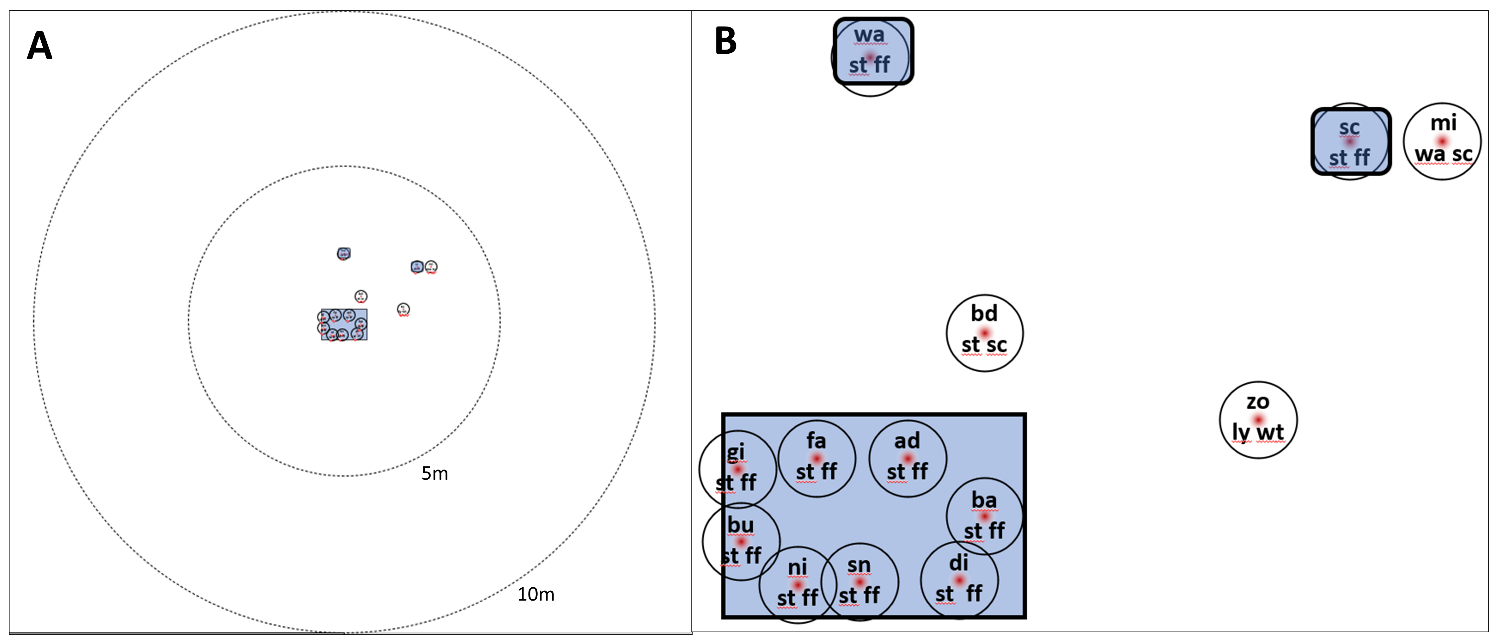
*

Supplemental Figure 1: Spatiobehavioural scan data were collected in specific powerpoint slides (one slide per scan) and then the coordinates and data exported as csv-files using VBA programming. Blue: feeding patches, white circles: individuals with codes for id, position (e.g. “st” for “standing”) and behaviour (e.g. “ff” for “feeding”). Slides were centred around the main food patch. (A) entire slide with circles for 5m and 10m distance from the centre of the main food patch, and (B) zoom-in into the record shown in (A).

Supplemental Figure 2: Winner-looser-matrix of dominance ranks (normalized David’s score, DS). Columns are winners, rows are losers, both sorted by decreasing DS. Directional Consistency Index: 0.90, p(h’) = 0.040, transitivity (scaled) = 0.96 (p < 0.001), 73.8 % unknown relationships.


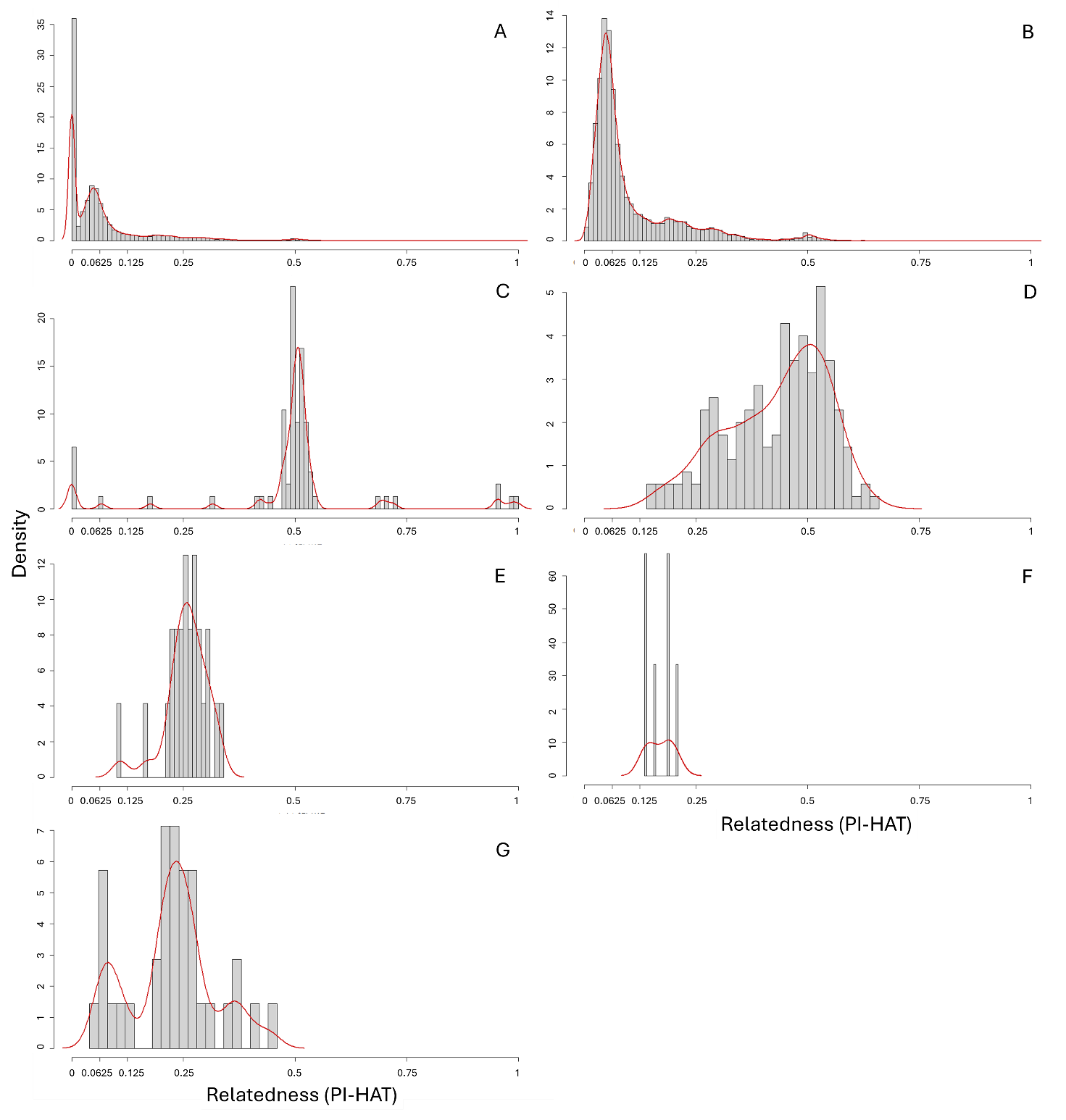


Supplemental Figure 3: Distribution of relatedness (PI-HAT) values of the entire sample population (density plots). (A) Entire population. (B) Entire population but PI-HAT = 0 removed for better visibility. (C) Known mother-offspring pairs. (D) Known siblings from the same litter. (E) Known grandparents. (F) Known great-grandparents. (G) Known cousins.


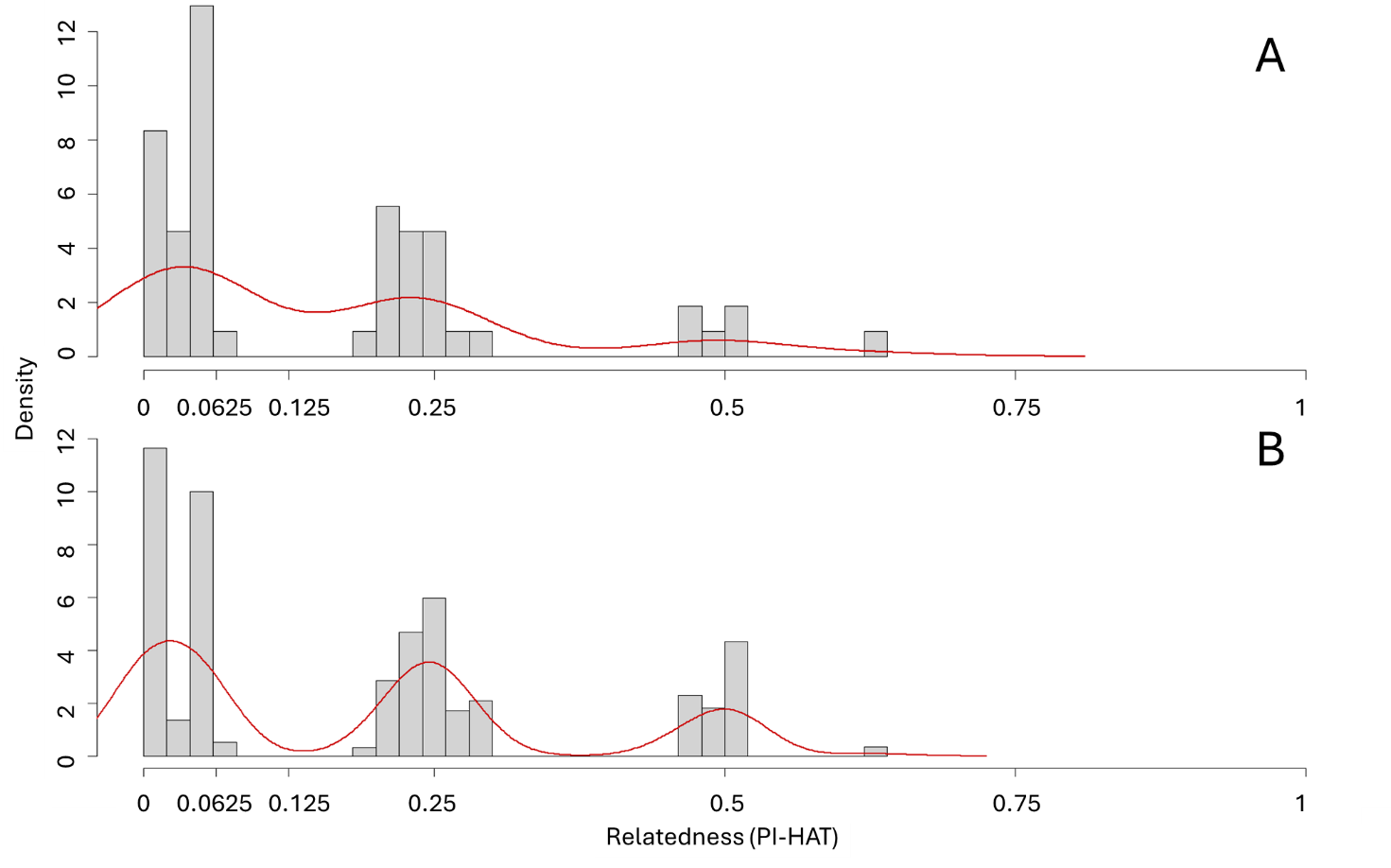


Supplemental Figure 4: Distribution of relatedness (PI-HAT) values of the feeding tests (density plots). (A) All dyads. (B) Distribution of all dyads present at the feeding sessions, across all scans.


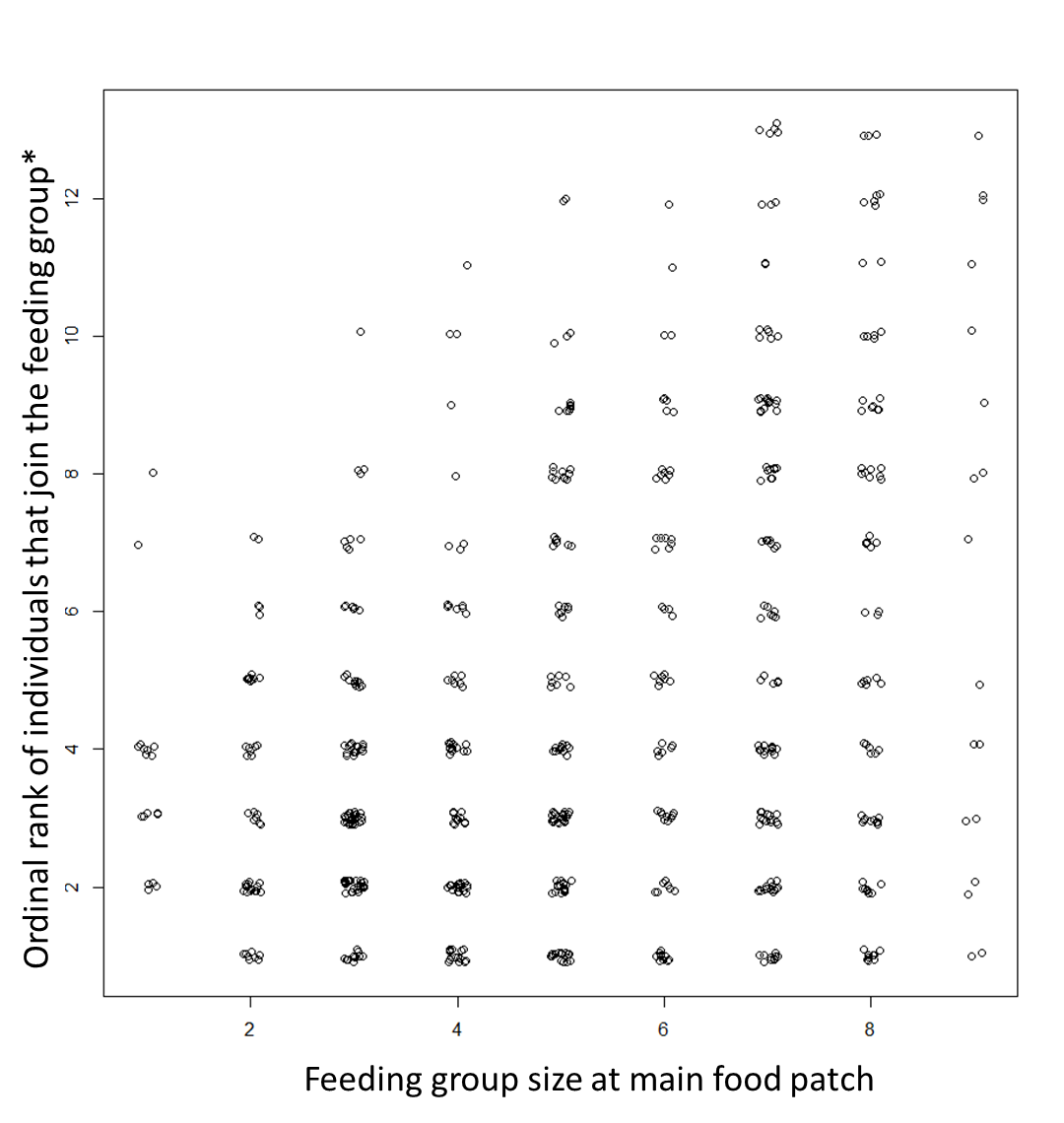


Supplemental Figure 5: Successively lower-ranking individuals join with increasing feeding group size. Data points slightly scattered (jittered) for better visibility. Higher rank number means lower dominance rank. *Rank among all feeding and non-feeding individuals that are present at a scan.

*Supplemental tables*

Supplemental table 1: Predictors of aggression rate by the two highest-ranking individuals at the main food patch, focusing on the CSI (left) and familiarity (right) instead of feeding group size (Model 8). Poisson GAMM on one value per scan. Aggression before: Number of aggression and dominance behaviours (interaction bouts) during the one minute between the preceding and the current scan. Aggression after: Number of aggression and dominance behaviours (interaction bouts) during the one minute between the current and the following scan. P-values in bold: Significant predictor variables.

Supplemental table 2: Predictors of aggression rate by all individuals at the main food patch. Poisson GAMM on one value per scan (Model 8). Aggression before: Number of aggression and dominance behaviours (interaction bouts) during the one minute between the preceding and the current scan. Aggression after: Number of aggression and dominance behaviours (interaction bouts) during the one minute between the current and the following scan. Right table: Without the potential outlier. P-values in bold: Significant predictor variables.

Supplemental table 3: Results of binomial GAMM on the probability to cofeed within 0.8 m head distance (instead of feed) in relation to rank effects and relatedness (Model 4). Dyadic values per scan. P-values in bold: Significant predictor variables.

Supplemental table 4: Ethogram

| Aggressive behaviour (low aggression) | Staring at: Actor stares at another subject, holding eye contact.  Barking: Actor barks at another subject.  Baring teeth: Actor bares its canines, or generally curls the lips.  Raising hackles: Subject raises the hackles.  Jaw spar: Two subjects "fencing" with open jaws.  Snapping: To snap teeth into the air, noisily.  Pointing at: The Actor stretches its body while it stares at another subject.  Lunging at: Actor moves abruptly and rapidly towards recipient, for a distance of 1 or a few meters, while staring at him/her but without making physical contact. |
| --- | --- |
| Aggressive behaviour (high aggression) | **Attacking:** Running into or jumping onto another with tail, ears and sometimes hackles up, often with bites at the neck.  **Knocking down:** To strike another subject sharply with the chest or shoulder so that the other loses balance and maybe falls to the ground.  **Pinning:** To grab another at the neck or at the muzzle, forcing it down to the ground and holding it there.  **Biting:** The dog bites another dog in the skin and fur.  **Fighting:** The subject and the receiver engage in reciprocal biting and aggressive physical contact.  **Chasing:** A subject runs after a conspecific, exhibiting threatening behaviours. |
| Dominance  behaviour | **Standing tall:** Subject straightens up to full height, with a rigid posture, may include raised hackles, ears erect and tail perpendicular or above the back. Can be standing but also during walking, especially towards the receiver.  **Standing over:** To stand over another's body, with all four paws on the ground. The receiver may have either the whole body or just the forepaws under the actors’ belly/side. With tail held high and a tense body posture.  **Paws on:** Actor approaches recipient from the side and puts one forepaw or both over recipient’s back; the actor's body posture being rigid.  **Riding up:** Actor places his forepaws around recipient’s torso, and he/she may or may not thrust his/her pelvis.  **Head on:** The subject approaches another’s shoulder/back/head and puts its head on it. Most of times formation looks like a capital “T”. Actor can either place his/her head over recipient without making body contact or can push recipient’s head downwards.  **Muzzle bite:** To grab the muzzle of another subject either softly or with enough pressure to make the other whimper. |
| Submissive  behaviour | **Avert gaze:** Actor turns his or her head away in response to the receiver either reducing the distance between, displaying dominant/aggressive behaviours, or orienting towards them.  **Head dip:** Actor lowers his/her head in response to the receiver either reducing the distance between, displaying dominant/aggressive behaviours, or orienting towards them. During the displaying of this behavioral pattern, the actor’s muzzle may point downwards or forwards but the head and neck are leaning downwards.  **Flattening ears:** Actor retracts the ears into the neck or holds them backwards in response to the receiver either reducing the distance between, displaying dominant/aggressive behaviours, or orienting towards them.  **Tail dip:** Actor lowers his/her tail in response to the receiver either reducing the distance between, displaying dominant/aggressive behaviours, or orienting towards them. The tail can be held near the hind legs or tucked between them.  **Crouching:** Lowered posture bending the legs, arching the back, lowering the tail between the hind legs.  **Fleeing:** To run away from another with tail tucked between the legs and body ducked.  **Belly exposure:** To lie on the back showing the stomach holding the tail between the legs. The ears are held back and close to the head and the subject can raise a hind leg for inguinal presentation.  **Withdrawing:** The subject creates a distance between itself and another subject, by moving away from it, but not at full speed.  **Avoidance:** The subject makes (the beginning) of a movement, away from another subject. Quite often, only the upper body is moved, the subject never moves over a large distance. Can also occur during the process of approaching another subject.  **Whimper:** Actor emits a high-pitched, plaintive vocalization in response to an aggressive or dominance signal. |
| Affiliative behaviour | **Approach:** The actor approaches another subject within one body length for at least 5 seconds, without necessarily showing interest in the other.  **Leave:** To move away from another subject, beyond one body length.  **Body contact:** Actor comes so close to another subject that they start to stay with at least a part of their bodies in contact and in a relaxed position.  **Grooming:** Starting to nip, lick or scratch the fur or skin occasionally the neck.  **Bow:** one subject crouches down touching or almost touching forelimbs to the ground with rear end high in the air, orientation is directed towards partner.  **Start Social Play:** Actor starts to engage with a subject in motor patterns such as bite, chase, run around one another, kick, jump and maybe snap or bite without enough pressure to cause injury.  **Lie friendly:** To lie on the back, tail-wagging, maybe kicking with the foreleg against another subject sometimes with open mouth.  **Stand friendly:** The subject stands relaxed with ears pointed forward, while another is approaching it or orienting/looking towards it.  **Approach friendly:** The subject moves towards the receiver in relaxed posture with ears pointed forward.  **Body rubbing:** To rub one’s body against any part of the receiver’s body.  **Nose-to-nose:** To have the nose less than 10 cm away from the partner’s nose  **Stand over relaxed:** To stand over another's body, with all four paws on the ground. The receiver may have either the whole body or just the forepaws under the actors’ belly/side. The tail is low/neutral and the body is relaxed.  **Social sniff:** To sniff another’s body part except its anogenital area. |
| Other behaviours | **Feeding:** The individual is ingesting or chewing food.  **Waiting**: The individual waits (stationary or moving) and has their head turned towards the food with a neutral stance, but does not eat.  Ignoring: The subject does not react to aggressive or sexual behaviour it gets from another subject. |
